# Dynamics of macroeconomic factor effects on food assistance program participation in the United States

**DOI:** 10.1371/journal.pone.0269442

**Published:** 2022-06-13

**Authors:** Oral Capps

**Affiliations:** Department of Agricultural Economics, Texas A&M University, College Station, Texas, United States of America; Newcastle University, UNITED KINGDOM

## Abstract

Using polynomial distributed lag (PDL) models, the impacts of macroeconomic factors relating to economic, financial, and sociological stress and designed to be short-run predictors of U.S. economic performance are identified and assessed concerning participation in key food assistance programs (SNAP, WIC, and NSLP). The econometric analysis covers the period October 1999 to September 2020. The impact of COVID-19 on participation in these programs also is quantified. Based on the parameter estimates obtained from the econometric PDL models, ex-ante forecasts of participation in the SNAP, WIC, and NSLP subsequently are made and evaluated over the period October 2020 to August 2021. The empirical results show that different sets of macroeconomic drivers affect participation levels across the respective food assistance programs. No macroeconomic factor is common across SNAP, WIC, and NSLP participation. Changes in macroeconomic conditions which influence SNAP, WIC and NSLP participation are not just contemporaneous but also affect participation levels anywhere from 1 month to 12 months later. Importantly, this research allows not only the determination of the macroeconomic factors which affect program participation but also allows the determination of the ability of the respective models to forecast program participation. As such, the Food and Nutrition Service will be in better position to assess program needs as well as to forecast program participation levels to minimize errors in the budgetary process.

## Introduction

The United States Department of Agriculture (USDA) through the Food and Nutrition Service (FNS) administers various food and nutrition assistance programs such as the Supplemental Nutrition Assistance Program (SNAP), the Special Supplemental Nutrition Program for Women, Infants, and Children (WIC), and the National School Lunch Program (NSLP). These programs not only provide targeted populations access to food, a healthy diet, and nutrition education but also serve as a safety net and protection against income losses and unexpected expenses.

We identify and assess macroeconomic factors, particularly economic, financial, and sociological stressors, linked to participation in the SNAP, WIC, and NSLP. The extant literature places emphasis primarily on the level of real income and on the unemployment rate as the key macroeconomic variables in affecting participation in domestic nutrition assistance programs (predominantly SNAP). But a key contribution of this research is to extend that focus to additional macroeconomic factors and provide the ability to predict participation in food assistance programs. Attention is centered on a set of economic indicators developed by the Conference Board, a non-governmental organization, designed to predict activity in the U.S. economy six to nine months in future (http://www.conference-board.org/data/bcicountry.cfm?cid=1).

The respective macroeconomic factors or economic indicators considered are the unemployment rate; the ratio of total consumer credit outstanding to disposable personal income; the Kansas City Financial Stress Index; the St. Louis Financial Stress Index; the number of initial claims for unemployment insurance; manufacturers’ new orders of durable goods and non-defense capital goods; real disposable personal income; real M2 money stock; housing starts; the Case-Shiller Home Price Index; and the University of Michigan Consumer Sentiment Index (consumer expectations about the economy). These macroeconomic variables are commonplace in economic studies and are easily obtained from the Federal Reserve Economic Database [1]. Because the impacts of these macroeconomic factors on participation in the SNAP, WIC, and NSLP may be distributed over time and not just contemporaneous, the use of polynomial distributed lag (PDL) models is adopted.

The principal goals of this research are fourfold: (1) to identify and assess the impacts of selected macroeconomic factors on participation in key food assistance programs (SNAP, WIC, and NSLP) administered by the FNS using PDL models; (2) to quantify the impact of COVID-19 on participation in the SNAP, WIC, and NSLP; (3) to forecast SNAP, WIC, and NSLP participation based on the use of the PDL models; and (4) to evaluate the forecast performance associated with the respective PDL models. This research allows not only the determination of the macroeconomic factors which affect program participation but also allows the determination of the ability of the respective models to forecast program participation. As such, the FNS will be in better position to assess program needs as well as to forecast program participation levels to minimize errors in the budgetary process.

We hypothesize that the impacts associated with the Kansas City Financial Stress Index; the St. Louis Financial Stress Index; the number of initial claims for unemployment insurance; the unemployment rate; and the ratio of total consumer credit outstanding to disposable personal income are positively related to the level of participation in the SNAP, WIC, and NSLP. These respective macroeconomic factors are economic stressors. As such, the greater the economic stress, the greater the likelihood of increased participation in food assistance programs. On the other hand, we hypothesize that the impacts associated with manufacturers’ new orders of durable goods and non-defense capital goods; real disposable personal income; real M2 money stock; housing starts; the Case-Shiller Home Price Index; and the University of Michigan Consumer Sentiment Index are negatively related to the level of participation in the SNAP, WIC, and NSLP. These macroeconomic factors often serve as predictors of U.S. economic performance. Consequently, with improved economic performance, the lower the likelihood of increased participation in food assistance programs.

## Background on U.S. food assistance program participation

**Fig 1:**
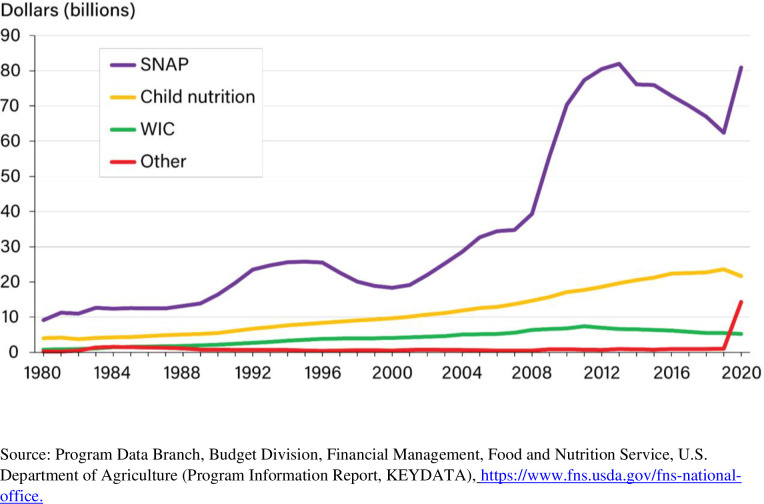
USDA expenditures for food assistance, fiscal 1980–2020.

The United States Department of Agriculture (USDA) is responsible for providing food and nutrition assistance through Food and Nutrition Service (FNS) programs [2] that include the Supplemental Nutrition Assistance Program (SNAP), the Special Supplemental Nutrition Program for Women, Infants, and Children, commonly known as WIC,and various child nutrition programs. Child nutrition programs include the National School Lunch Program (NSLP), the School Breakfast Program (SBAP), the Child and Adult Care Food Program (CACFP), the Summer Food Service Program (SFSP), and the Fresh Fruit and Vegetable Program (FVVP). Established on August 8, 1969, FNS is the government agency responsible for administering U.S. domestic nutrition assistance programs. Nominal USDA expenditures for food assistance from fiscal years 1980 to 2020 are exhibited in Fig 1. On the basis of Fig 1, these expenditures for food assistance have been increasing over time. So, reducing forecasting errors is especially important now to reduce the likelihood of budgeting misallocations. Because the SNAP, the NSLP, and the WIC constitute the bulk of the respective USDA expenditures for food assistance, in this study, we center attention on these programs. Specifically, our interest lies with the monthly level of participation in each of these food assistance programs from fiscal years 1999 to 2020.

### Level of SNAP participation

Under federal rules, to be eligible for participation for the SNAP, housholds must meet tests associated with their gross monthly income, net income, and assets. Household income before or after deductions are applied must be at or below the poverty line, and assets must fall below certain limits. Federal poverty levels are used to determine eligibility and benefits for food assistance programs. The poverty levels vary by family size and by household income.

**Fig 2:**
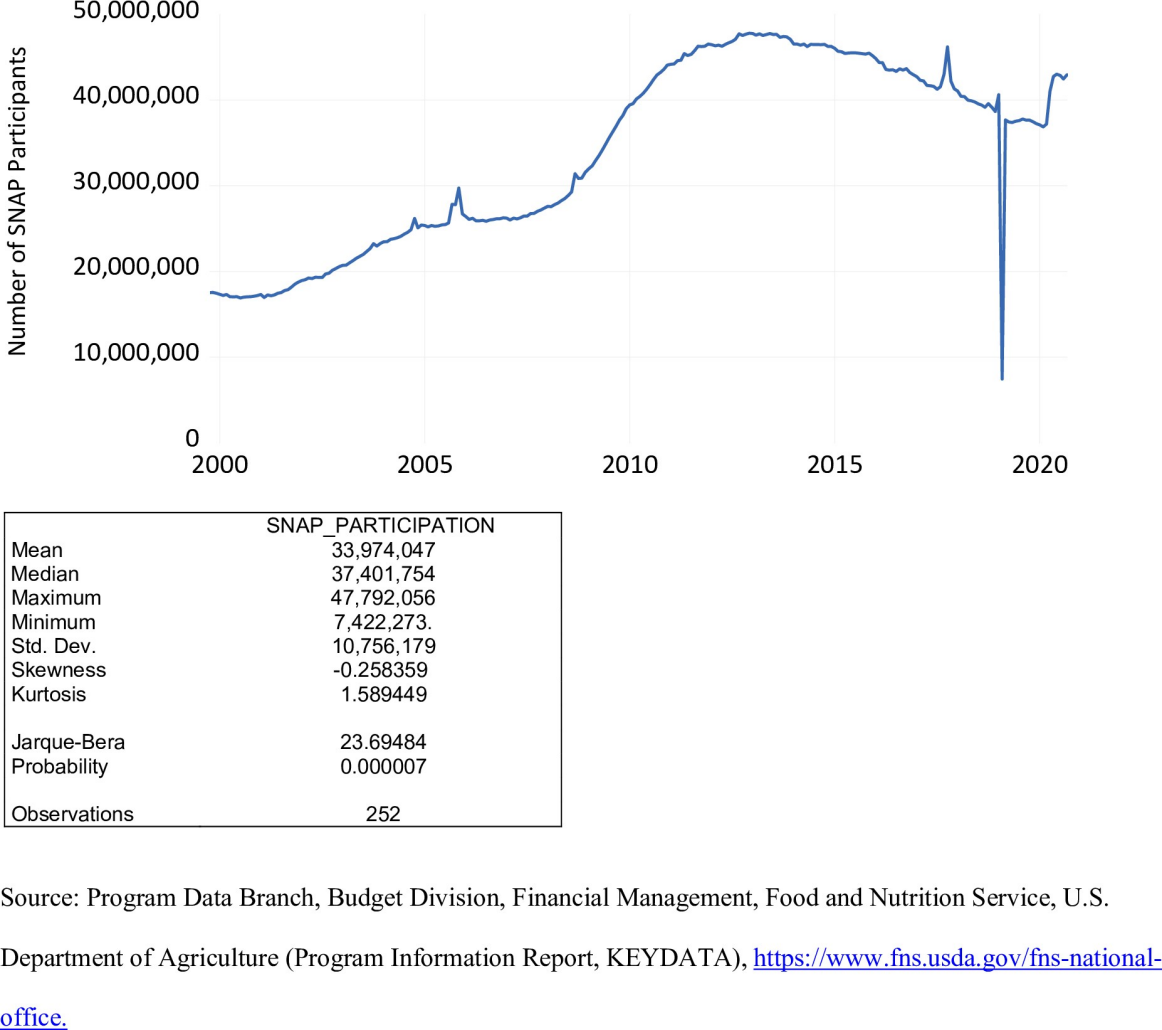
SNAP participation, October 1999 to September 2020.

The level of SNAP participation over the period October 1999 to September 2020 is exhibited in Fig 2. The number of SNAP participants during this period ranged from 7,422,273 (February 2019) to 47,792,056 (December 2012), averaging 33,974,047. Pre-COVID 19 defined as the period October 1999 to March 2020, the level of SNAP participation for fiscal year 2020 averaged 33,766,003. Post-COVID 19 defined as the period April 2020 to September 2020 in this study, the level of SNAP participation averaged 42,503,879. The precipitous decline in SNAP participation that occurred in Februray 2019 was due to the partial government shutdown that began in late December 2018. Based on the Jarque-Bera statistic, the level of SNAP participation is not normally distributed. The issue of non-normality affects the appropriate confidence internal associated with predictions. Typically, analysts often derive a 95% confidence interval by combining the forecast plus or minus two standard errors. Because participation levels in the SNAP, WIC, and NSLP are not normally distributed, this method no longer yields a 95% confidence interval.

### Level of WIC particpation

**Fig 3:**
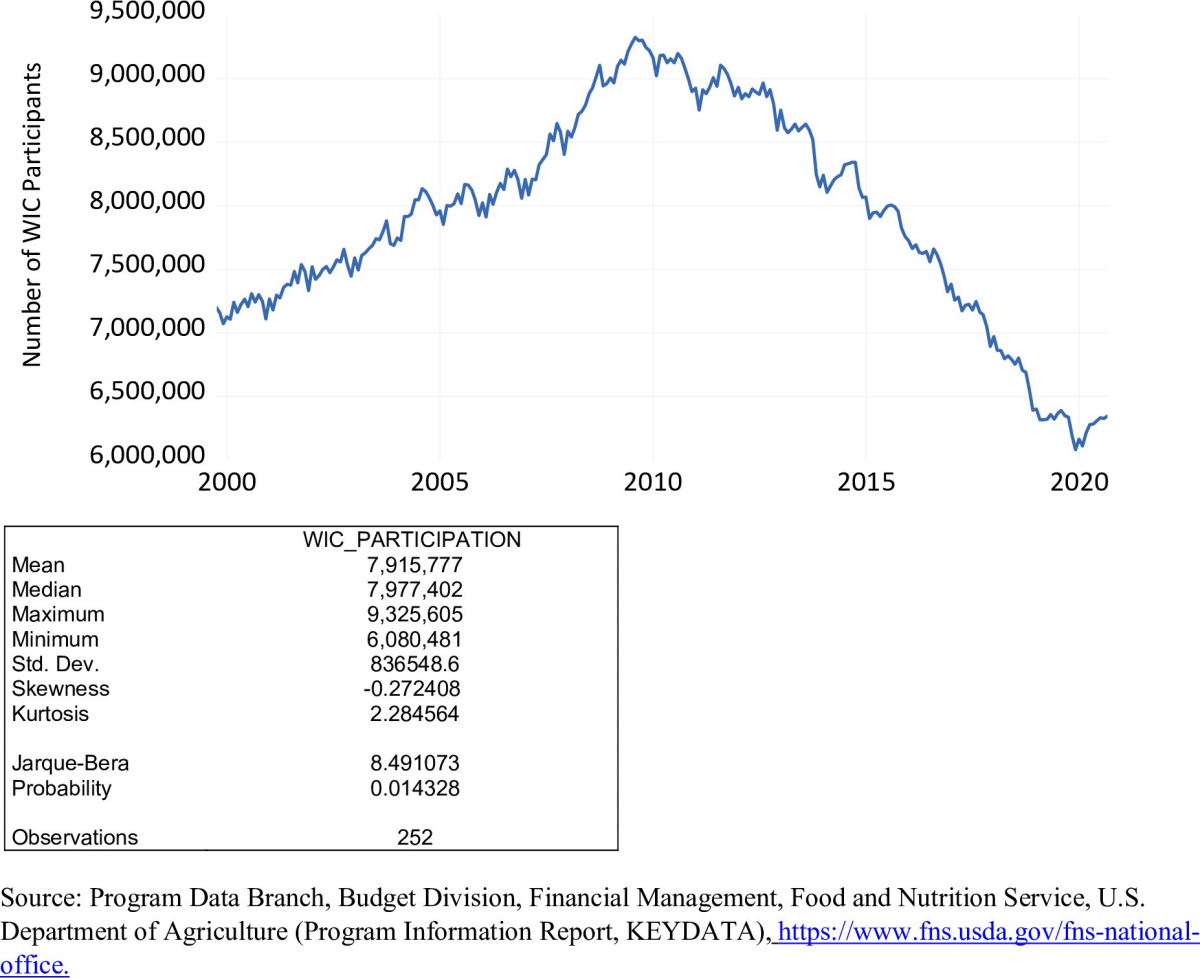
WIC participation, October 1999 to September 2020.

To be eligible for participation in the WIC program, the income of any applicant must be between 100 percent and 185 percent of the federal poverty guidelines issued each year by the Department of Health and Human Services. Certain applicants can be determined income-eligible based on their participation in certain programs such as the SNAP, the Temporary Assistance for Needy Families (TANF) program, and the Medicaid program. The level of WIC participation, that is, the total number of women, infants, and children over the period October 1999 to September 2020 in the program, is exhibited in Fig 3. The number of WIC participants during this period ranged from 6,080,481 to 9,325,605, averaging 7,915,777. Pre-COVID 19, the level of WIC participation averaged 7,954,905. Post-COVID 19, the level of WIC participation averaged 6,311,513. WIC participation was on the rise from October 1999 to August 2009. Subsequently, WIC participation was on the decline from September 2009 until the presence of the COVID-19 pandemic. Based on the Jarque-Bera statistic, the level of WIC participation is not normally distributed.

### Level of NSLP participation

**Fig 4:**
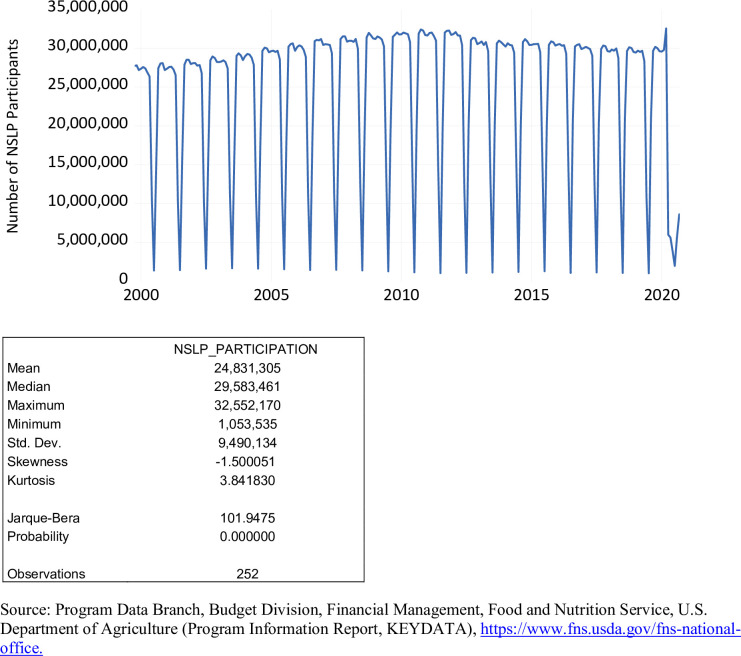
NSLP participation, October 1999 to September 2020.

To qualify for free meals in the NSLP, household income must be either income below 130 percent of the poverty level or meet qualifications for the SNAP or the TANF. To qualify for reduced-price meals in the NSLP, household income must be between 130 and 185 percent of the poverty line. The level of NSLP participation over the period October 1999 to September 2020 is exhibited in Fig 4. The number of NSLP participants during this period ranged from 1,053,535 to 32,552,170 averaging 24,831,305. Pre-COVID 19, the level of NSLP participation averaged 25,307,881. Post-COVID 19, the level of NSLP participation averaged 5,291,707. Without question, seasonality is evident with NSLP participation due to the summer months of June, July, and August. NSLP participation is lowest in the month of July. Based on the Jarque-Bera statistic, the level of NSLP participation is not normally distributed.

### Data on macroeconomic factors

We identify and assess the impacts of the twelve aforementioned macroeconomic factors considered to be economic, financial, and sociological stressorslinked to participation in the SNAP, WIC, and NSLP. The frequency of the set of macroeconomic variables in this analysis is monthly in order to match the frequency of SNAP, WIC, and NSLP participation. The descriptive statistics, figures, and the ensuing econometric analysis associated with the aforementioned macroeconomic factors cover the period October 1999 to September 2020. Of note, the pairwise correlation coefficients for this set of factors ranges from -0.7370 to 0.9819.

**Fig 5:**
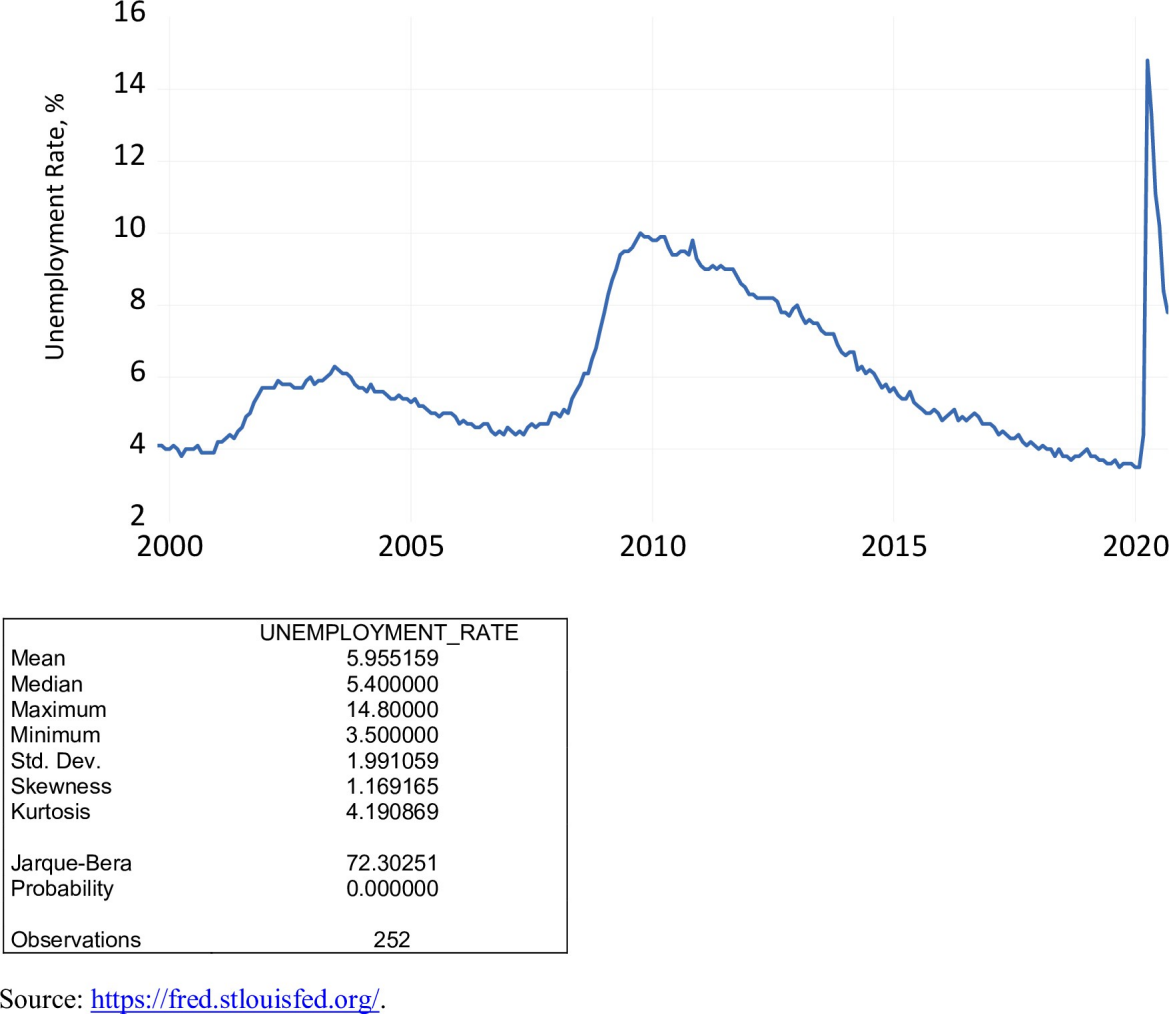
The unemployment rate, percent, October 1999 to September 2020.

The unemployment rate (UNRATE) represents the number of unemployed as a percentage of the labor force. Labor force participation is restricted to those 16 years of age and older, who currently reside in 1 of the 50 states or the District of Columbia, who do not reside in institutions (e.g., penal and mental facilities, homes for the aged), and who are not on active duty in the Armed Forces. The unemployment rate is a seasonally-adjusted series. As exhibited in Fig 5, over the period October 1999 to September 2020, the unemployment rate varied from 3.5 percent to 14.8 percent, averaging close to 6 percent. During the COVID-19 pandemic, the unemployment rate ranged from 7.8 percent to 14.8 percent, averaging 10.9 percent.

**Fig 6:**
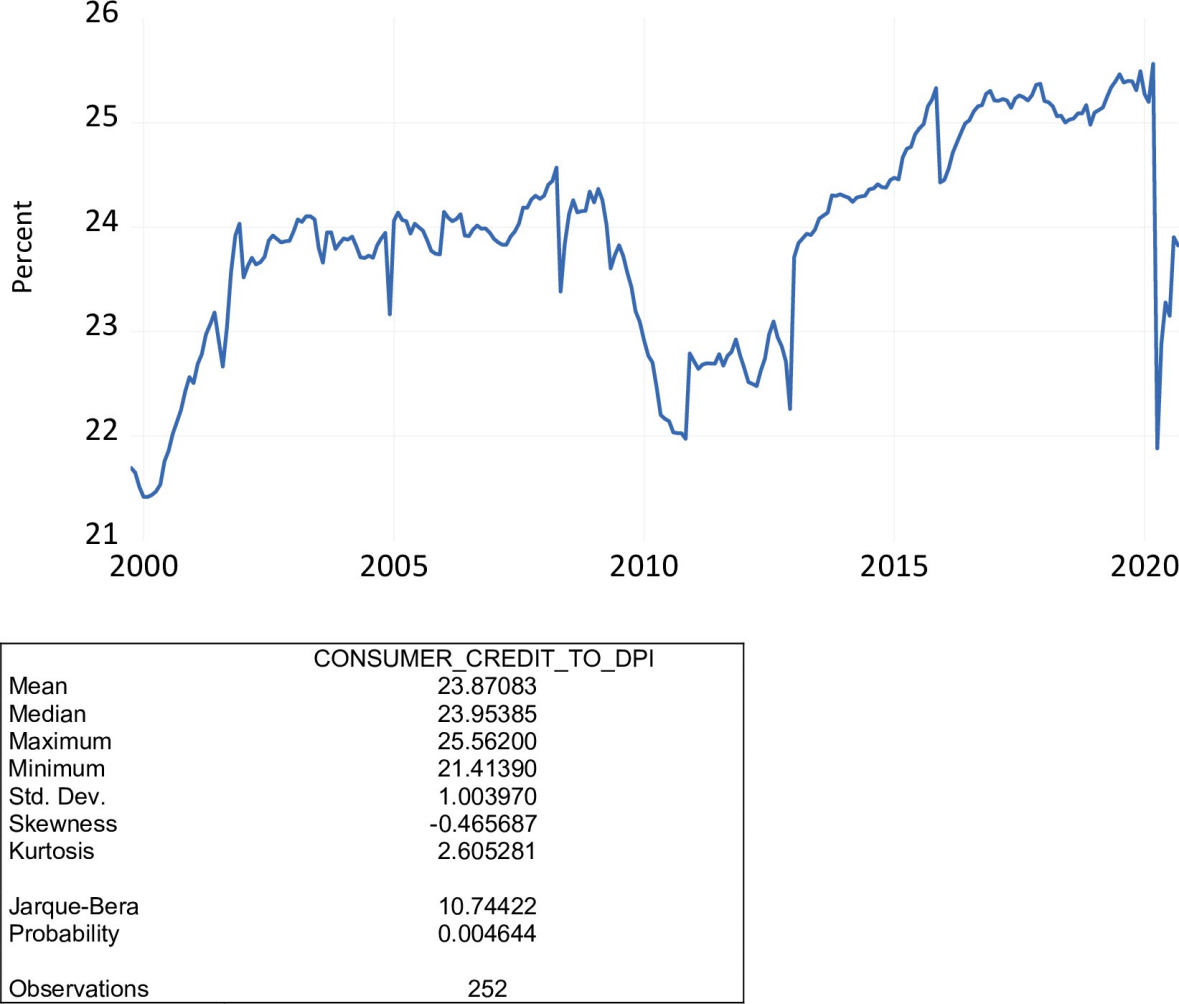
The ratio of consumer credit outstanding to disposable personal income, percent, October 1999 to September 2020.

We combine the seasonally-adjusted series of total consumer credit owed and scrutinized outstanding (TOTALSL) with the seasonally-adjusted series of disposable personal income (DSPI) to form a ratio. This ratio is expressed as a percent of disposable personal income. As shown in Fig 6, over the period October 1999 to September 2020, the ratio of total consumer credit outstanding to disposable personal income ranged from 21.41 percent to 25.56 percent, averaging 23.87 percent. During the COVID-19 pandemic, the ratio of consumer credit owed to disposable personal income ranged from 21.88 percent to 23.90 percent, averaging 23.15 percent.

The Kansas City Financial Stress Index (KCFSI) is a composite index of 11 variables reflecting stress in the U.S. financial system, compiled by the Kansas City Federal Reserve Bank. These variables fall into two broad categories: average yield spreads and behavior of asset prices. The index is calculated using a principal components procedure [3]. Variables included in this financial stress index are: (1) 3-month London Interbank Offered Rate (LIBOR)/Treasury Bill spread; (2) 2-year swap spread; (3) off-the-run/on-the-run 10-year Treasury spread; (4) Aaa/10-year Treasury spread; (5) Baa/Aaa spread; (6) High-yield bond/Baa spread; (7) consumer asset backed securities (ABS)/5-year spread; (8) correlation between returns on stocks and Treasury bonds; (9) implied volatility of overall stock prices or stock market volatility (VIX); (10) idiosyncratic volatility (IVOL) of bank stock prices; and (11) cross-section dispersion (CSD) of bank stock returns.

**Fig 7:**
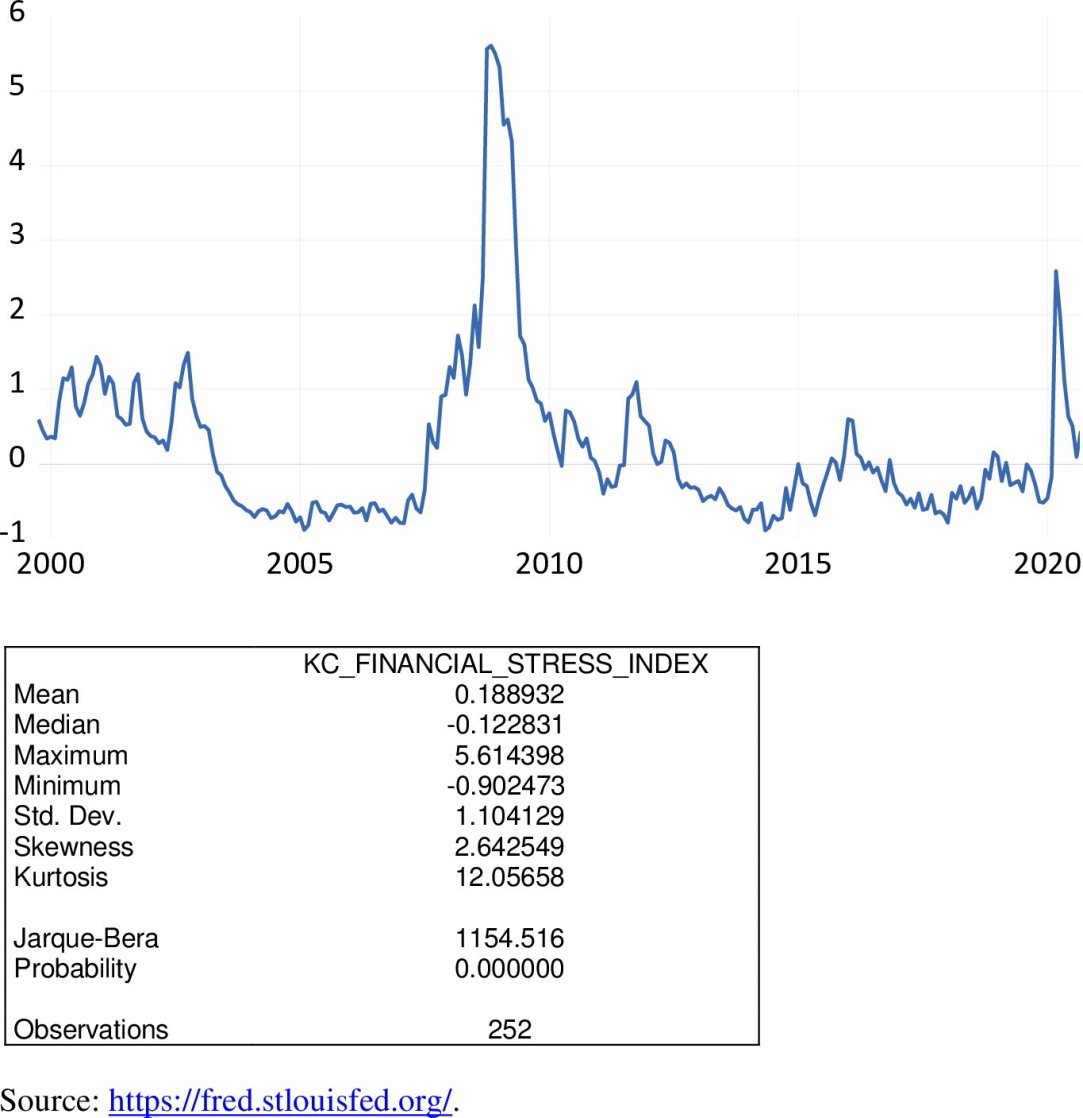
The Kansas City finanical stress index, October 1999 to September 2020.

As exhibited in Fig 7, over the period October 1999 to September 2020 the KCFSI varied from -0.9025 to 5.6144. During the COVID-19 pandemic, the KCFSI ranged from 0.0876 to 1.9783, averaging 0.7923. A positive value of the KCFSI indicates that financial stress is above the long-run average, while a negative value signifies that financial stress is below the long-run average. The financial stress during the time of the Great Recession was much higher than during the COVID-19 pandemic.

The St. Louis Financial Stress Index (STLFSI) is similar to the construction of the KCFSI. This index is a composite index of 18 variables which deal broadly with interest rates (e.g. federal funds rate, 2-year Treasury, 10-year Treasury, and 30-year Treasury), yield spreads (e.g. 10-year Treasury minus 3-month Treasury, 3-month commercial paper minus 3-month Treasury bill, and corporate Baa-rated bond minus 10-year Treasury), and other financial indicators (e.g. Chicago Board Options Exchange Market Volatility Index, Merrill Lynch Bond Market Volatility Index and J.P. Morgan Emerging Markets Bond Index). Each of the 18 components of the STFSI captures some aspect of financial stress [4]. Similar to the KCFSI, principal component analysis also is used to construct the STLFSI.

**Fig 8:**
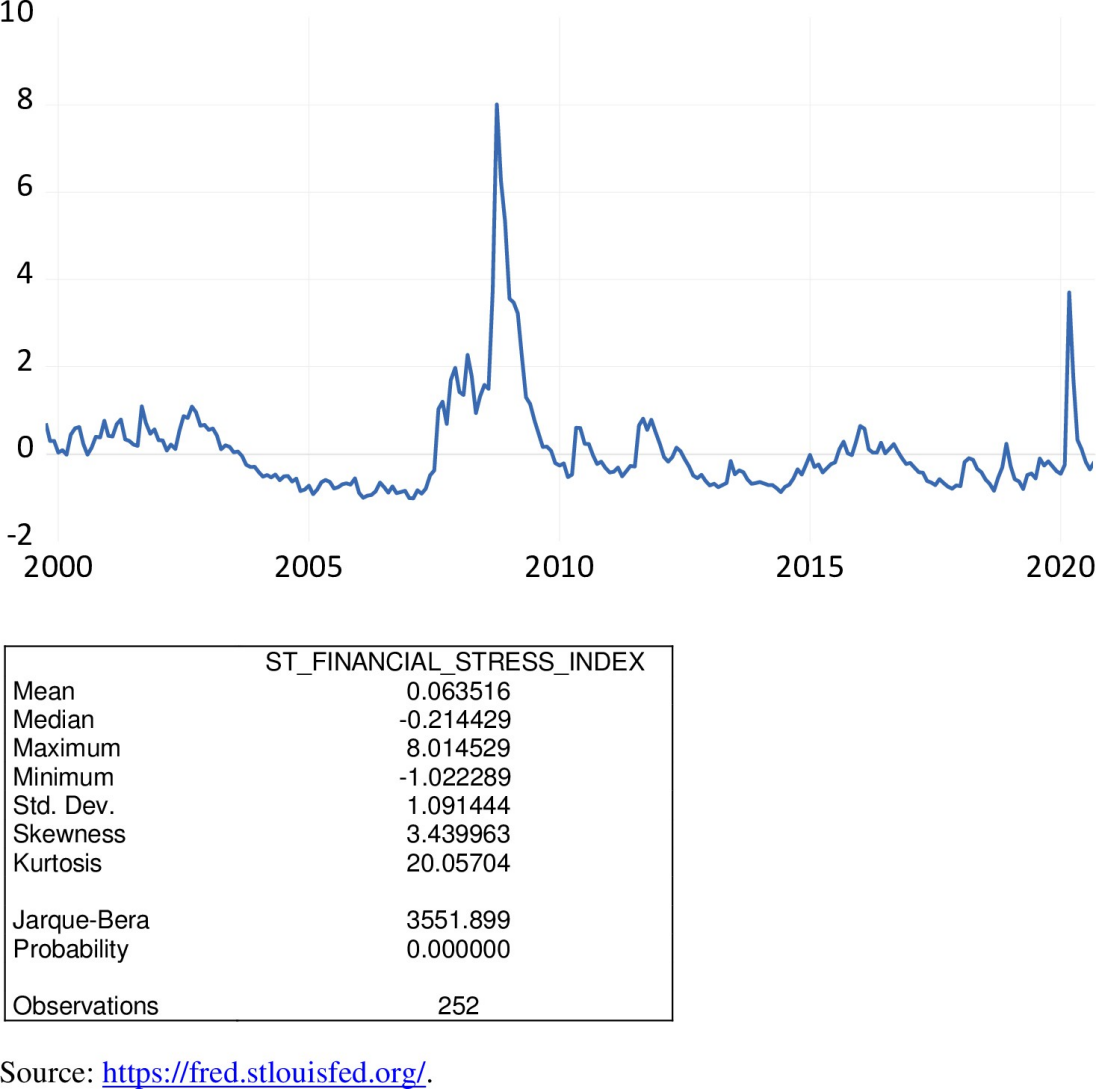
The St. Louis finanical stress index, October 1999 to September 2020.

As exhibited in Fig 8, over the period October 1999 to September 2020, the STLFSI ranged from -1.0223 to 8.0145, averaging 0.0635. During the COVID-19 pandemic, the STLFSI ranged from -0.3604 to 1.7164 averaging 0.2315. A positive value of the STLFSI indicates that financial stress is above the long-run average, while a negative value signifies that financial stress is below the long-run average. The financial stress during the time of the Great Recession was much higher than during the COVID-19 pandemic.

While the KCFSI is available on a monthly basis, the STLFSI is available on a weekly basis. As such, it was necessary to transform this weekly series to a monthly series to maintain the same data frequency. Neither of these indices is seasonally adjusted. The correlation coefficient associated with these two financial stress indices is 0.9239; hence the KCFSI and the STLFSI move together.

**Fig 9:**
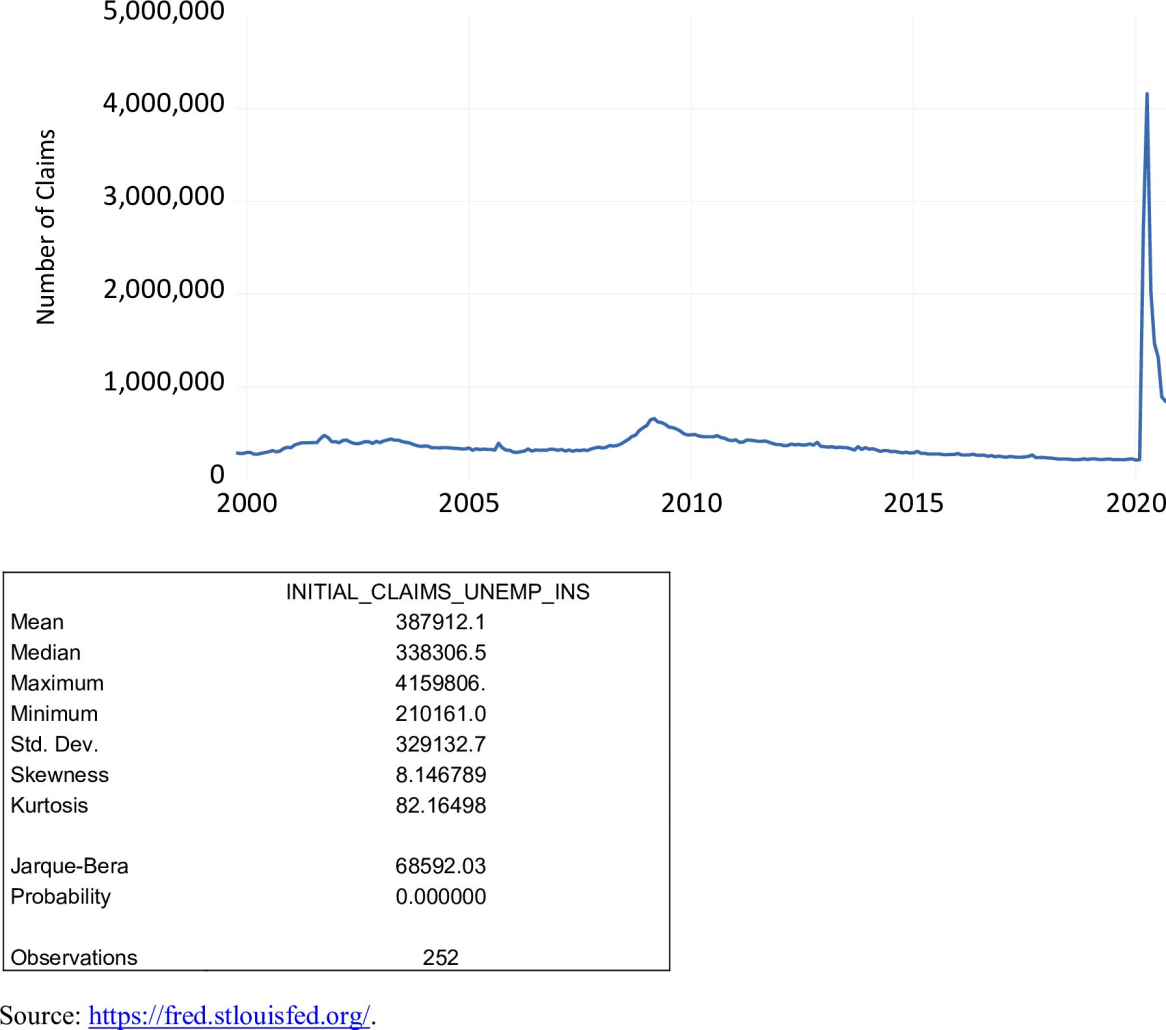
Initial claims of unemployment insurance, October 1999 to September 2020.

The initial claims for unemployment insurance (ICSA) serves as a measure in economic analysis of unemployment trends in the United States. The ICSA is available on a weekly basis and is seasonally adjusted. Similar to the STLFSI, it was necessary to transform this series to a monthly series to maintain the same data frequency. As shown in Fig 9, over the period October 1999 to September 2020, ICSA varied from 210,161 to 4,159,806 averaging 387,912. During the COVID-19 pandemic, the ICSA ranged from 839,800 to 4,159,806, averaging 1,783,447.

**Fig 10:**
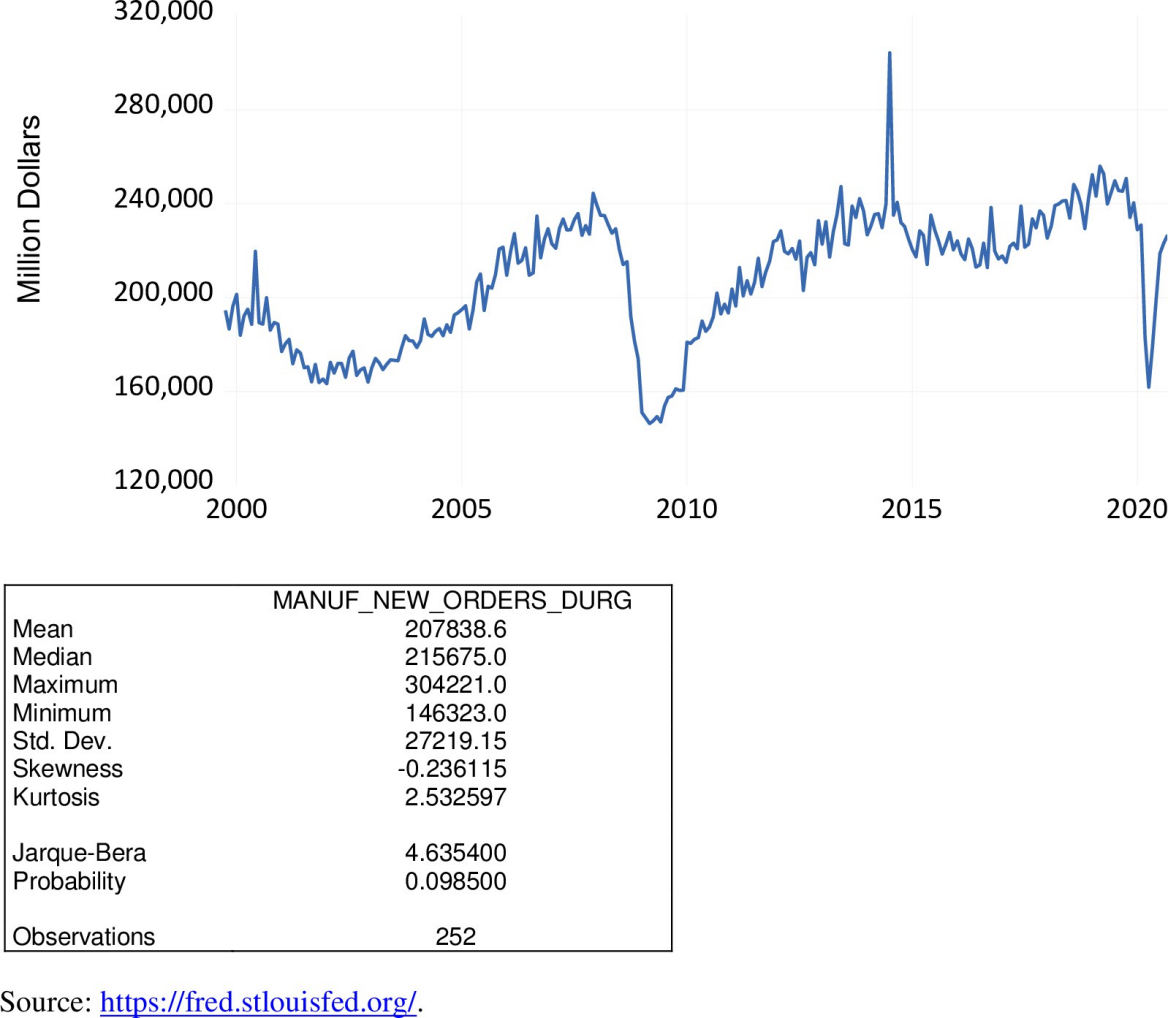
Manufacturers’ new orders of durable goods, million $, October 1999 to September 2020.

Manufacturers’ new orders of durable goods is a measure of current industrial activity, a key economic indicator. These data come from a broad-based monthly survey conducted by the U.S. Census Bureau. Higher values of manufacturers’ new orders of durable goods indicate that the U.S. economy is on the upswing, while lower values indicate a downward trajectory. As shown in Fig 10, over the period October 1999 to September 2020, new orders of durable goods varied from $146.323 billion to $304.221 billion, averaging $207.839 billion. During the COVID-19 pandemic, new orders of durable goods ranged from $161.833 billion to $226.664 billion averaging $201.416 billion.

**Fig 11:**
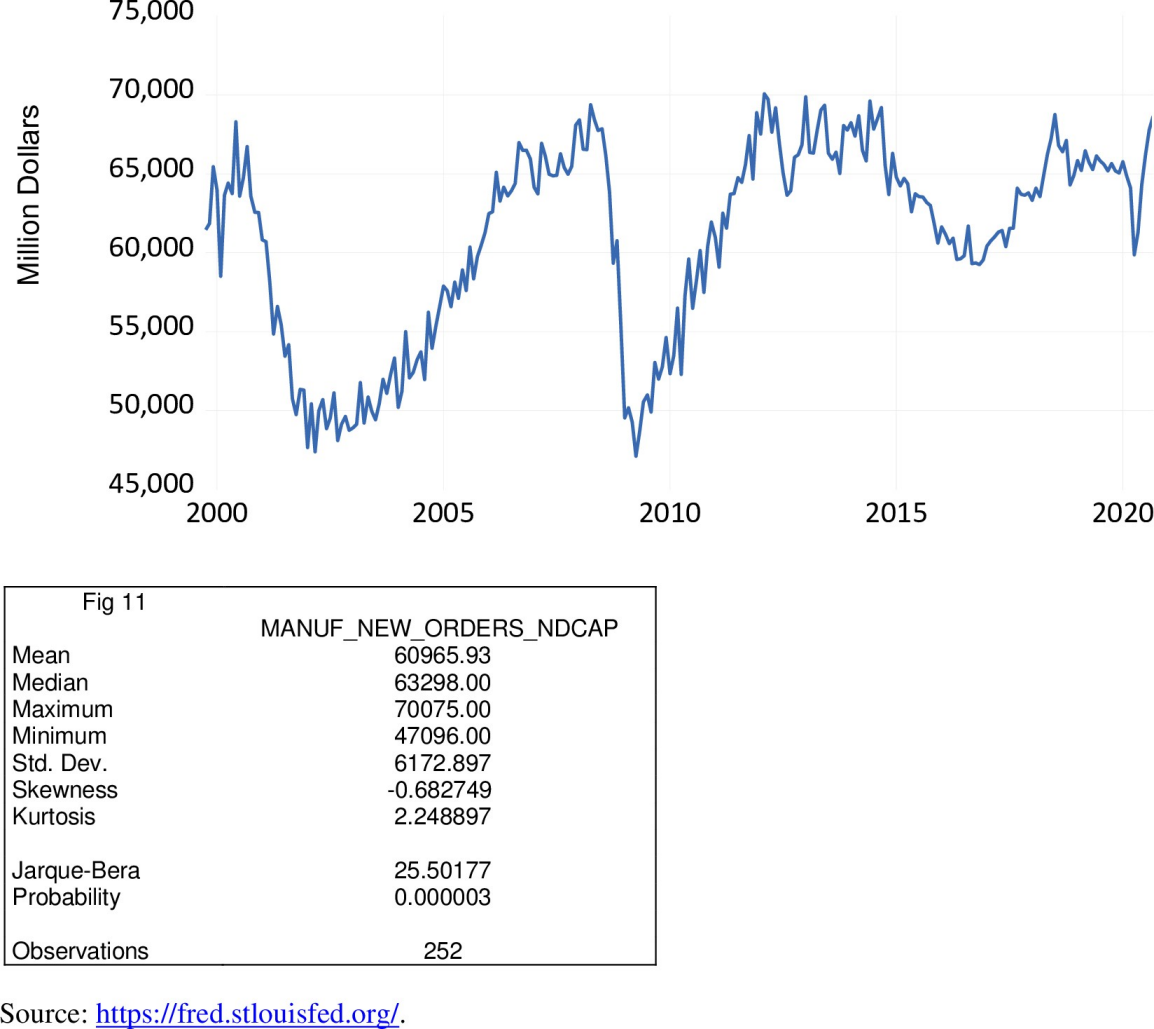
Manufacturers’ new orders of non-defense capital goods, million $, October 1999 to September 2020.

Manufacturers’ new orders of non-defense capital goods include among other things farm machinery and equipment; construction machinery; computers; turbines, generators, and other power transmission equipment; communications equipment; heavy duty trucks; office and institutional furniture; and medical materials and supplies. This macroeconomic variable provides broad-based data on economic conditions in the domestic manufacturing sector. Similar to manufacturers’ new orders of durable goods, these data come from a broad-based monthly survey conducted by the U.S. Census Bureau. Higher values of manufacturers’ new orders of non-defense capital goods are supportive of a rise in the U.S. economy, while lower values are supportive of a decline. As shown in Fig 11, over the period October 1999 to September 2020, new orders of non-defense capital goods ranged from $47.096 billion to $70.075 billion, averaging $60.966 billion. During the COVID-19 pandemic, the values of manufacturers’ new orders of non-defense capital goods ranged from $59.872 billion to $68.646 billion, averaging $64.683 billion.

Both measures of manufacturers’ new orders are seasonally adjusted. The correlation associated with these two series is 0.8491. As such, these macroeconomic factors exhibit a strong linear and positive association.

**Fig 12:**
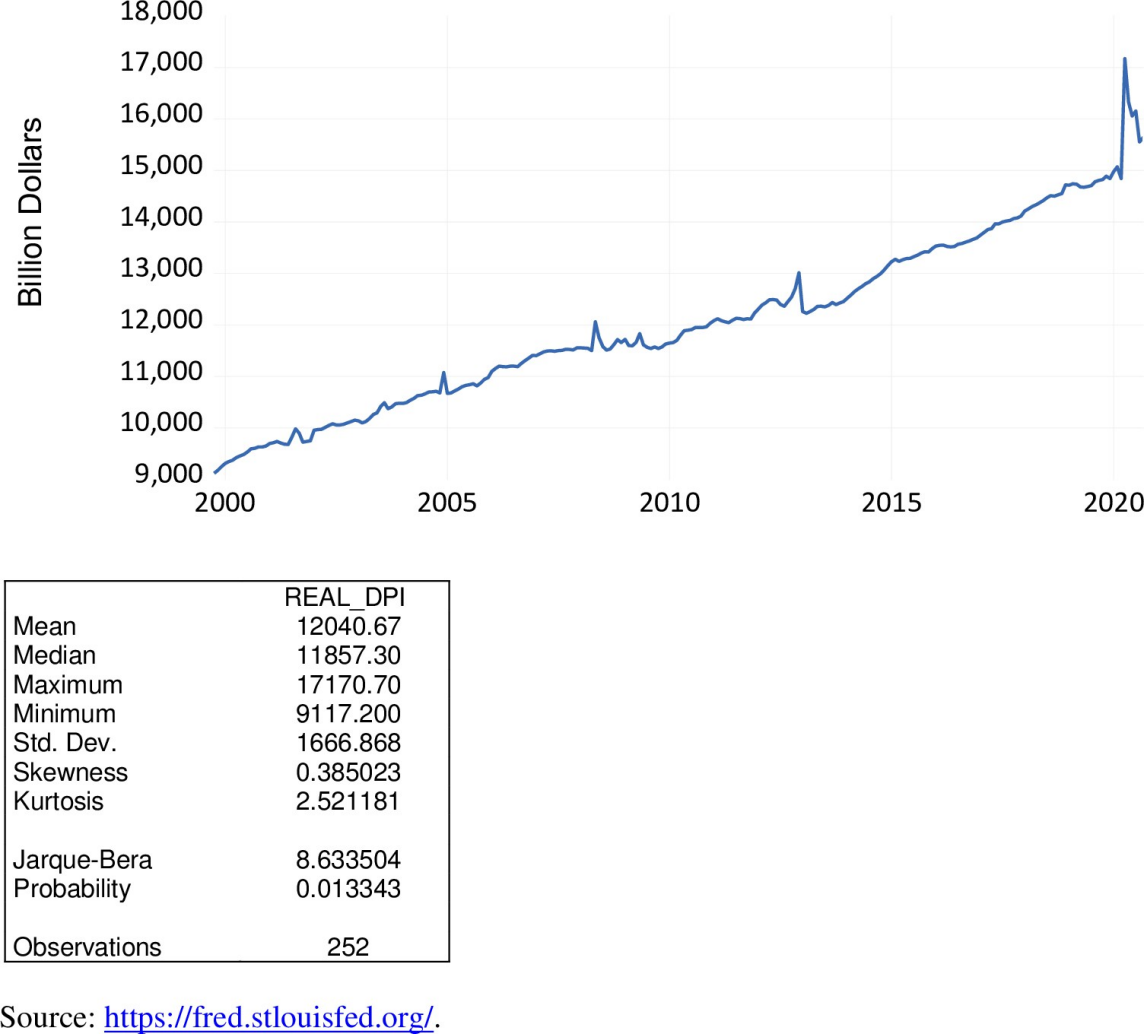
Real disposable personal income, billion $, October 1999 to September 2020.

Disposable personal income is the amount of money left after income taxes have been deducted. This measure is closely monitored as a key economic indicator to gauge the overall state of the economy. To account for inflation, this measure of income is divided by the Consumer Price Index. Hence, in this analysis we employ real or inflation-adjusted disposable personal income. As well, this macroeconomic measure has been seasonally adjusted. As exhibited in Fig 12, over the period October 1999 to September 2020, real disposable personal income varied from $9.117 trillion to$17.171 trillion, averaging $12,041 trillion. Since reaching its peak in April 2020, this measure has been on the decline from May 2020 to September 2020.

M2 is a measure of the money supply that includes cash, checking deposits, savings deposits, money market securities, and mutual funds. M2 as a measurement of the money supply is a critical factor in the central banking (monetary) policies of the Federal Reserve System. This measure has been adjusted for inflation. Hence, similar to the use of real disposable personal income, in this analysis we employ real or inflation-adjusted M2. This measure also has been seasonally adjusted.

**Fig 13:**
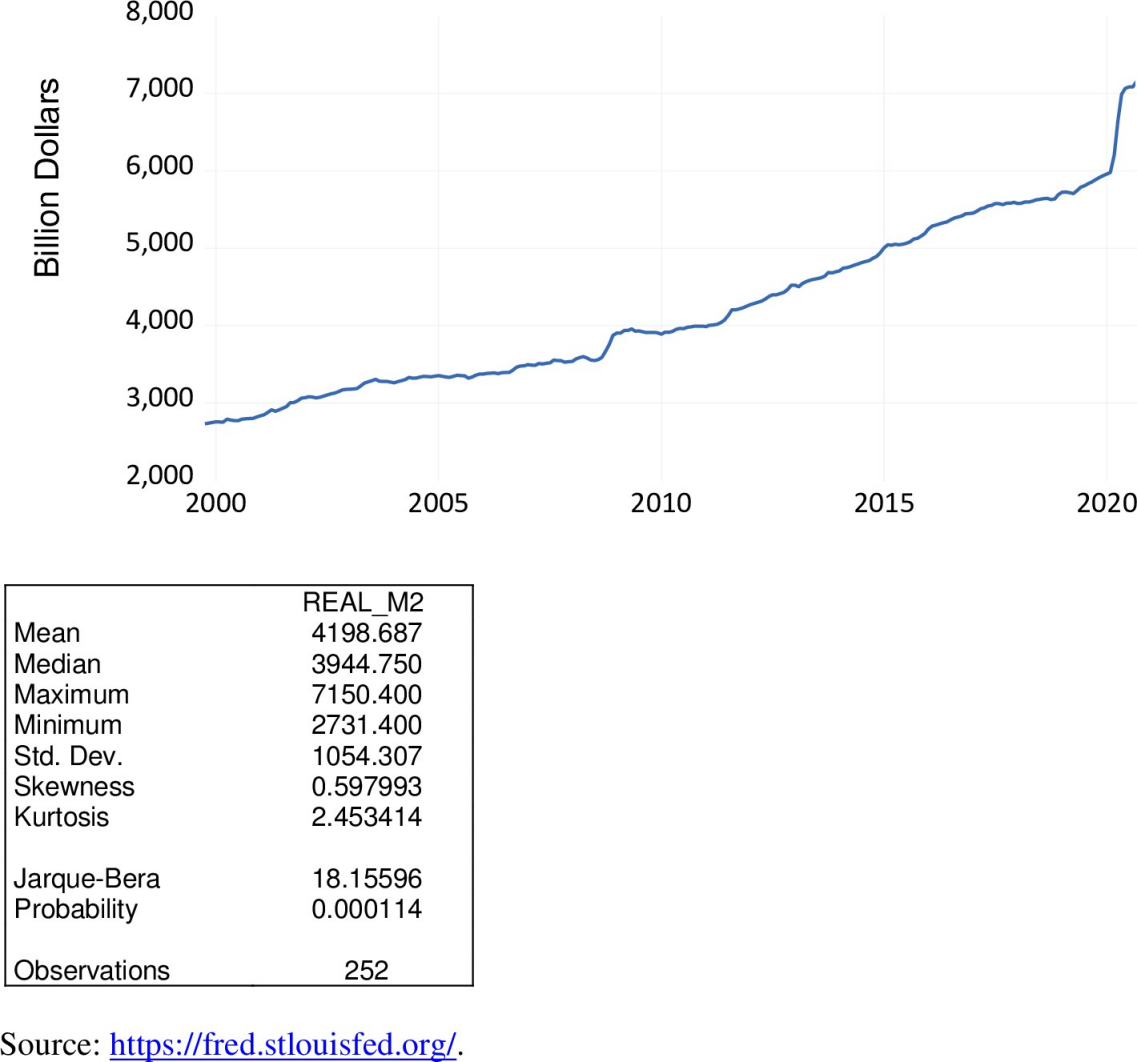
Real M2 money stock, billion 1982–84 $, October 1999 to September 2020.

As exhibited in Fig 13, over the period October 1999 to September 2020, real M2 ranged from $2.731 trillion to $7.150 trillion, averaging $4.199 trillion. While this macroeconomic measure has risen monotonoically over the period October 1999 to September 2020, the magnitude of real M2 has accelerated since the beginning of the COVID-19 pandemic from $6.651 trillion to $7.150 trillion.

**Fig 14:**
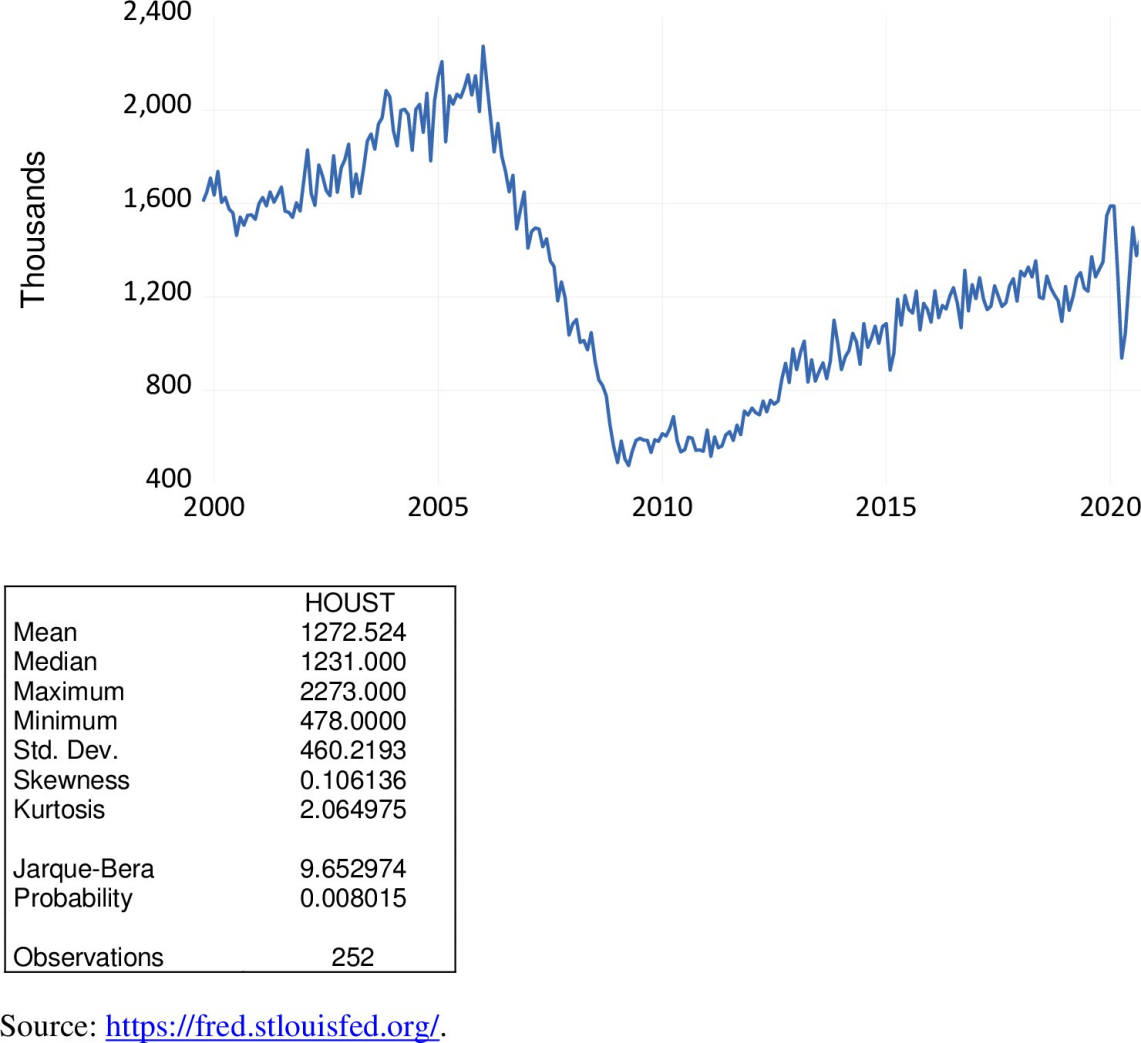
Housing starts, thousands, October 1999 to September 2020.

Housing starts reflects the number of privately owned new houses (housing units) on which construction has been started. Housing starts consist of single-family houses, townhouses or small condos, and apartment buildings with five or more units. This macroeconomic variable is a key economic indicator of the U.S. economy. As exhibited in Fig 14, over the period October 1999 to September 2020, housing starts, seasonally adjusted, ranged from 478,000 to 2,273,000, averaging 1,272,524. During the COVID-19 pandemic, housing starts ranged from 938,000 to 1,497,000, averaging 1,263,000. Values of this measure were below average during the April 2020 to June 2020, the initial months of the COVID-19 pandemic. Since June 2020, housing starts have rebounded.

The S&P Case–Shiller U.S. National Home Price Index is a composite of single-family home price indices for the nine U.S. Census divisions. Based on work by economists Karl Case and Robert Shiller, this measure is calculated monthly using a three-month moving average. The S&P national index is normalized to have a value of 100 for January 2000. This index is the leading measure of U.S. residential real estate prices.

**Fig 15:**
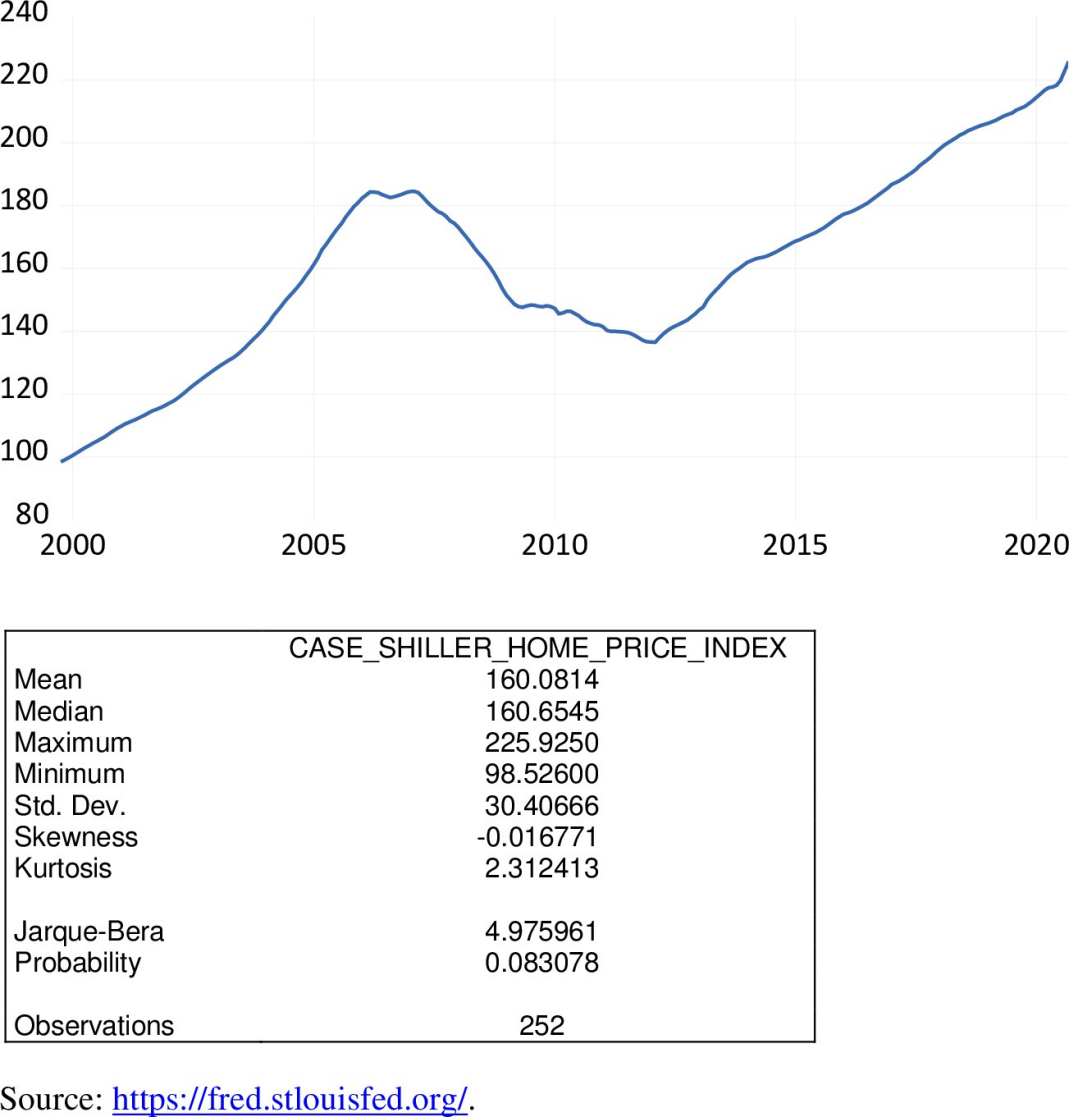
The S&P case-shiller home price index, January 2000 = 100, October 1999 to September 2020.

As exhibited in Fig 15, over the period October 1999 to September 2020, the Case-Shiller home price index ranged from 98.526 to 225.925, averaging 160.081. Values of this measure have continued its monotonic rise even during the COVID-19 pandemic. This measure is not seasonally adjusted.

The Consumer Sentiment Index is a consumer confidence index published monthly by the University of Michigan. The index is normalized to have a value of 100 in December 1966. This index is based on a nationally-representative survey of consumers who are asked 50 questions focusing on their own financial situation, the short-term nature of the U.S. economy, and the long-term nature of the U.S.economy.

The Consumer Sentiment Index was created and still is published with the following objectives: to assess near-time consumer attitudes on the business climate, personal finance, and spending; to promote an understanding of, and to forecast changes in, the national economy; to provide a means of incorporating empirical measures of consumer expectations into models of spending and saving behavior; to gauge the economic expectations and probable future spending behavior of the consumer; and to judge the consumer’s level of optimism/pessimism. Each month at least 500 telephone interviews are conducted from a continguous U.S. sample.

**Fig 16:**
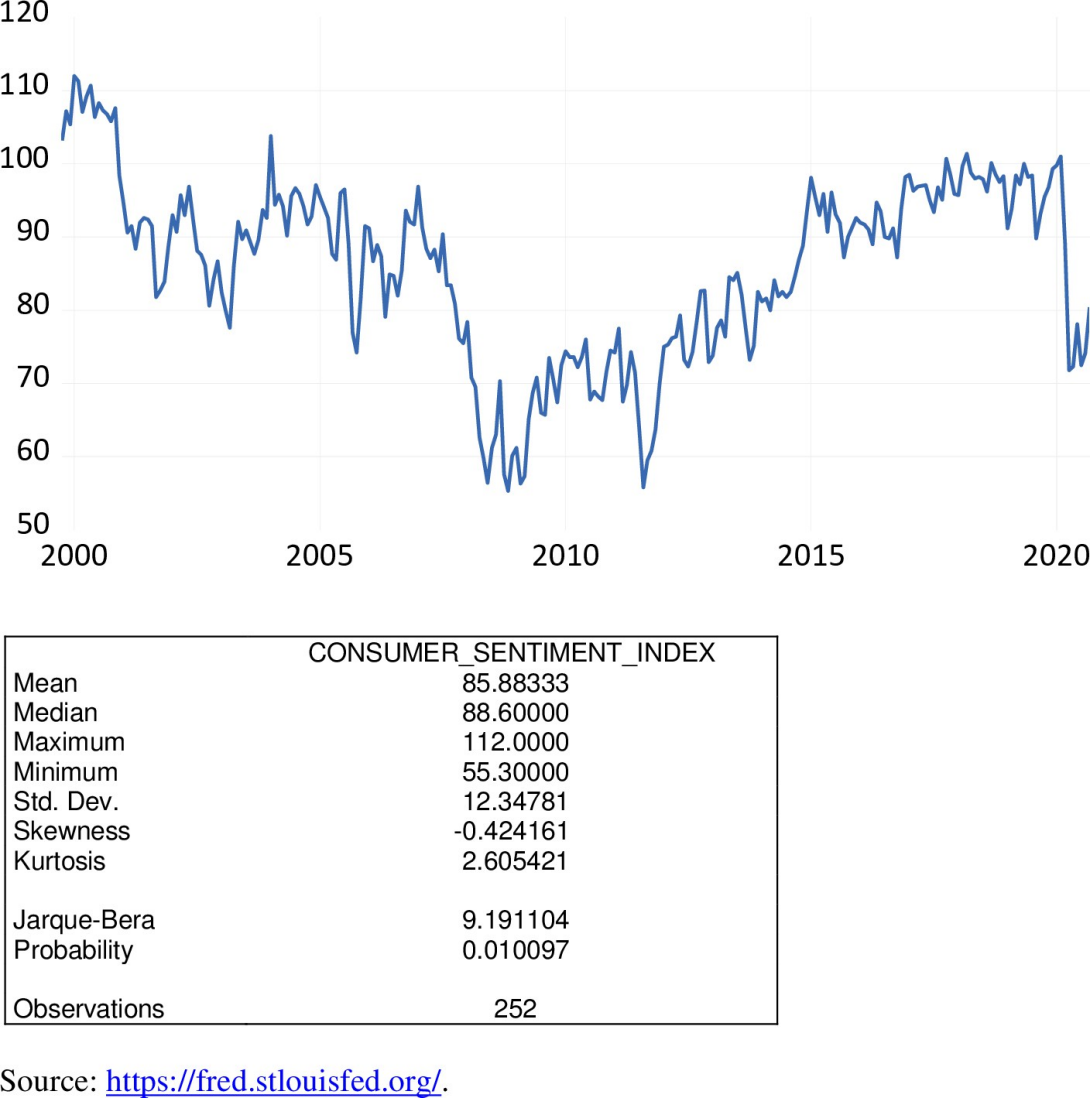
Consumer sentiment index, 1966 quarter 1 = 100, October 1999 to September 2020.

As exhibited in Fig 16, over the period October 1999 to September 2020, the Consumer Sentiment Index varied from 55.3 to 112.0, averaging 85.9. Values of this measure declined noticeably during the COVID-19 outbreak. During the COVID-19 pandemic, the Consumer Sentiment Index ranged 71.8 to 80.4, averaging 74.9. This measure is not seasonally adjusted.

## Review of literature

Extensive research evidence substantiates that SNAP functions effectively as a fiscal stabilizer, meaning that as the economy and market incomes fall during recessions participation in SNAP rises and as market incomes rise during economic expansions participation falls [5–12]. Ziliak [12] suggested that the greatest factor underlying the increase in SNAP participation after the Great Recession, and indeed since 2000, was the weak macroeconomy characterized by higher unemployment, lower income, and widening income inequality.

The literature on the relationship of SNAP participation with economic conditions dates back to work by Wertheimer and Fletcher [13] and Dynaski, Rangarajan, and Decker [14], wherein national quarterly data were used to estimate the impact of program legislation in conjunction with the effects of the unemployment rate and real wages on SNAP participation. In particular, over the period 1977 to 1988 Wertheimer and Fletcher [13] found that increases in the unemployment rate increases SNAP participation and that decreases in real wages are associated with increases in SNAP participation.

The Personal Responsibility and Work Opportunity Reconciliation Act (PRWORA), the landmark 1996 welfare reform legislation replacing Aid to Families with Dependent Children (AFDC) with Temporary Assistance to Needy Families (TANF), prompted studies to center attention on factors affecting SNAP caseloads [15]. To illustrate, according to Currie and Grogger [16] decreases in the unemployment rate contributed to declines in SNAP caseloads (SNAP participation). Gleason et al. [17] noted the link between the economy and the decline in SNAP caseloads from 1994 to 1999. Hanson and Gunderson [18] in examining the link between unemployment and SNAP participation, found that a one-percentage point increase in the unemployment rate led to a rise in SNAP caseloads by 700,000 in the short run and 1.3 million in the long run. Similarly Kornfeld [19] documented the effects of changes in the unemployment rate on SNAP caseloads during the period 1987–1999. Farrell et al. [20], in considering the role of the dynamics of household income in determining SNAP participation, found an inverted-U relationship between current household income and participation rates, holding other household characteristics constant. In addition, McKernan and Ratcliffee [21] examined the relationship between SNAP participation and employment using data from the 1990 and 1996 panels of the Survey of Income and Program Participation (SIPP).

Clarke, Levedahl, and Reed [22] analyzed the relationship between SNAP caseloads and the macroeconomy using annual state-level panel data for the period 1980–1999. Mabli, Martin, Castner [23] documented that increases in the the unemployment rate led to increases in state SNAP caseloads over the period 2000 to 2006. Mabli and Ohls [24], using data from the SIPP over the period 2001 to 2003, examined how long-term instability in employment affected the association between employment transitions and the decision to enter and exit SNAP. Bottom line, whether at the state level or at the national level, changes in macroeconomic conditions as measured by the unemployment rate and/or real income are consistent with changes in SNAP caseloads.

Dickert-Conlin, Fitzpatrick, and Tiehen [15] centered attention on SNAP policies over twenty-five years with monthly state-level administrative data to measure program participation and the timing of state policy implementation. The basis of the work was on the use of counterfactual experiments to examine changes in SNAP caseloads under various assumptions about the re-adoption of federally uniform SNAP policies.

Newman [25] extended this discussion to other nutrition assistance programs in examining the implications of income volatility for participation in the NSLP. Hanson and Oliveira [26] investigated the relationship between economic conditions and participation in five nutrition assistance programs Supplemental Nutrition Assistance Program (SNAP), Special Supplemental Nutrition Program for Women, Infants, and Children (WIC), National School Lunch Program (NSLP), School Breakfast Program (SBP), and Child and Adult Care Food Program (CACFP). The results of this study, based on annual data from 1976–2010, suggested that economic conditions influence participation in all the major nutrition assistance programs. The principal economic condition considered was the unemployment rate.

In investigating factors behind changes in SNAP participation, previous studies have relied upon aggregate caseload data [9–11, 27–29], program administrative microdata [30, 31], or household survey data [11, 12, 32–34]. The extant literature places emphasis primarily on the level of real income, the volatility of income, and the unemployment rate as the key macroeconomic variables in affecting participation in domestic nutrition assistance programs. Unlike previous studies, we center attention on a granular array of other macroeconomic variables besides real income and the unemployment rate. Other factors may have more predictive ability than these commonly used macroeconomic variables.

Additionally, this study involves a more recent time period, October 1999 to September 2020, spanning 21 fiscal years. We also expand coverage to WIC and NSLP participation levels and not just center attention on SNAP particpation levels. Unlike past studies, this research rests on monthly data rather than annual data, cross-sectional data, or panel data. As well, for the first time in the extant literature, this analysis deals with the use of polynomial distributed lags in econometric models to allow for dynamics of macroeconomic factors in affecting levels of participation in the SNAP, the WIC Program, and the NSLP. Finally, unlike previous studies, we forecast SNAP, WIC, and NSLP participation from October 2020 to August 2021 and evaluate the forecast performance of the aforementioned polynomial distributed lag models. Hence, we add measurably to the extant economic literature on this topic. Notably however we do not consider the level of participation in the SBP, the CACFP or in other FNS programs, and we do not consider the level of participation in various states or regions. That said, our work can be extended to these areas in future endeavors.

## Methodology

Structural/econometric models are developed and estimated to shed light on interrelationships among various macroeconomic variables and participation in the SNAP, the WIC, and the NSLP. With the exception of the Kansas City and St. Louis Financial Stress Indices, we use logarithmic transformations of all non-discrete variables in the analysis. Because these financial indices may take on negative values, the use of a logarithmic transformation is not possible mathematically. To account for the participation in USDA food assistance programs, we consider the monthly number of participants in the SNAP, the WIC, and NSLP for the United States. Similar to the work by Hanson and Oliveira (2012), participation figures are not normalized by population and reflect the actual number of participants. The analysis covers the period October 1999 to September 2020 which spans 21 fiscal years. This time frame includes behavior before and after the Great recession which occurred in 2008/2009, any policy changes in the respective programs, and behavior before and during the COVID-19 pandemic.

The structural/econometric specification for this analysis is given as np_*it*_ = *f*(*M*F_1*t*_, *M*F_2*t*_,….,*M*F_*jt*_)+*ϵ*_*it*_, where np_it_ denotes the number of participants in the ith food assistance program in time period t, MF_jt_ refers to the jth macroeconomic factor in time period t, and *ϵ*_*it*_ corresponds to the stochastic error term associated with food assistance program i in time period t. To capture dynamics, polynomial distributed lags formulations of the aforementioned macroeconomic variables are entertained in order to determine short-run impacts and cumulative impacts of these factors on participation levels.

Changes in the macroeconomic factors considered are not felt just contemporaneously but are also distributed over time. Consequently, the use of polynomial distributed lag (PDL) models is adopted. As such, PDL models capture not only short-run impacts but also cumulative impacts of macroeconomic factors on participation in the SNAP, WIC, and NSLP. Polynomial degrees of orders two and three, with and without endpoint constraints, and lag lengths up to 12 periods were considered with degrees-of-freedom and the principle of parsimony in mind. A key issue in the use of distributed lag models is to empirically find the optimal lag length. The selection of the appropriate distributed lag model associated with the number of participants in the SNAP, the WIC Program, and the NSLP rests on the use of the Akaike, Schwarz, or Hannan-Quinn information criteria (AIC, SIC, HQC). Based on these criteria, models with the longer lag lengths of up to 24 months were not statistically superior to models with lag lengths of up to 12 months.

To mitigate collinearity issues, we consider only a single macroeconomic variable at a time in each of the respective model specifications. In this way, we are in position to isolate and discern the impacts and to evaluate the forecast ability of each macroeconomic factor concerning the number of participants in the SNAP, the WIC Program, and the NSLP.

For each macroeconomic variable with respect to each of the food and nutrition assistance programs, we estimate 48 different econometric models (lag lengths from 1 to 12, and polynomial degree of orders two and three, with and without endpoint constraints). Given the three food and nutrition assistance programs and given the twelve macroeconomic variables, in total, we estimate 1,728 econometric/structural models. Statistical tests were conducted to determine whether the endpoint (head and tail) restrictions hold. In all cases, statistical evidence supported the presence of both head and tail restrictions.

We control for seasonality of program participation through the use of monthly dummy or indicator variables or by seasonally-adjusting program participation. Further, we allow for inertia or stickiness in response of macroeconomic factors on participation through the use of lagged dependent variables (lags of the number of participants). Moreover, we capture the impact of the COVID-19 pandemic on SNAP, WIC, and NSLP participation through the use of a dummy variable, 1 from April 2020 to September 2020, and 0 otherwise. In March 2020, the World Health Organization (WHO) officially declared COVID-19 to be a pandemic.

To be sure changes in program policy occur through legislative changes and changes in administrative practices pertaining to such factors as eligibility rules, benefit levels, and outreach [26]. Consequently, changes in program policies may influence the relationship between program participation and the set of macroeconomic factors considered. We capture these effects directly through the use of indicator variables and indirectly through the examination of the behavior of the residuals in the respective econometric/structural models.

Finally, once the appropriate distributed lag model is determined, we provide ex-post forecasts of SNAP, WIC, and NSLP participation from October 2020 to August 2021. In order to construct these forecasts, the data pertaining to the macroeconomic variables and the data pertaining to the number of participants in the SNAP, WIC, and NSLP were updated from October 20202 through August 2021. To get some idea of the predictive performance of the respective models, we provide out-of-sample measures of forecast accuracy associated over the period October 2020 through August 2021. This assessment is done based on mean absolute percent error (MAPE), a common metric in forecast evaluation.

## Econometric models

The dependent variables in the ensuing structural/econometric models correspond to the level of participation in SNAP, WIC, and NSLP. We capture the disruption in the pattern of SNAP participation attributed to the partial government shutdown in December 2018 through the use of dummy variables (or indicator variables) for January 2019, February 2019, and March 2019. The rationale for the three dummy variables in the SNAP models is to capture anticipatory effects associated with the government shutdown in January 2019, to measure the impact of the government shutdown in February 2019, and to capture the posterior effects of the government shutdown in March 2019. Additionally, in the SNAP models we seasonally adjust participation levels, and we use indicator variables to capture changes in eligibility.

We capture the initial rise and subsequent decline in WIC participation with the use of trend variables (a linear trend and a quadratic trend). We capture the seasonal nature of program participation with the use of monthly dummy variables in the WIC and NSLP models. Although the seasonal adjustment transformation was considered for the WIC models and the NSLP program mdoels, it was not adopted based on empirical results and forecast performance. In all models, to account for inflation, we use real measures of manufacturers’ new orders of durable goods and non-defense capital goods, disposable personal income, M2, and the Case-Shiller Home Price Index.

To capture dynamics, distributed lags formulations of the aforementioned macroeconomic variables are entertained in order to determine short-run impacts and cumulative impacts of these factors on participation in food assistance programs. We use of polynomial distributed lags (Almon lags) in this endeavor [35–38]. Finally, we provide measures of out-of-sample predictive performance, the acid test of forecast ability, associated with the national models of participation in the respective food assistance programs. This assessment is done based on absolute percent error [39]. In practical terms, if FNS were to set its budget using insights from this analysis, reductions in forecast error translate to cost savings.

The estimation of the respective models rests on the use of the software package EVIEWS 11.0 [40]. We only report the econometric analysis of the statistically significant macroeconomic factors for each of the three previously discussed food assistance programs. The level of significance chosen for this study is 0.05. Hence, p-values less than 0.05 are indicative of the statistical significance of the estimated coefficients in the respective models. That is to say, the estimated coefficients associated with the set of macroeconomic variables are statistically different from zero when the associated p-values are less than 0.05.

### Empirical results for SNAP participation analysis

The estimated econometric models associated with SNAP participation are exhibited in Table 1. The dummy variables D2004M11, D2005M09, D2005M11, D2005M12, D2008M09, and D2017M11 reflect changes in program eligibility and/or administration. Their estimated coefficients indicate that these changes result in changes in SNAP participation anywhere from 7 percent to 11 percent in either direction. The residuals move as an autoregressive moving average process of order 1 (ARMA(1,1)). To account for the ARMA(1,1) process associated with the residuals, we use generalized least squares (maximum likelihood) to obtain estimates of the coefficients in each of the respective models. The signs of the coefficients associated with the macroeconomic factors are in accord with prior expectations. The significance of the one-month lag of the dependent variable indicates the presence of inertia or habitual behavior in the level of SNAP participation.

**Table 1:** Estimated econometric models associated with SNAP participation, October 1999 to September 2020.

	Macroeconomic Variable				
Explanatory Variable	Manufacturers’ New Orders of Durable Goods	St. Louis Financial Stress Index	Housing Starts	Kansas City Financial Stress Index	Initial Claims of Unemployment Insurance
	*Estimated Coefficient*				
**Partial Government Shutdown**					
D2019m01 (1 if January 2019; 0 otherwise)	0.0747	0.0719	0.0725	0.0717	0.0761
D2019m02 (1 if February 2019; 0 otherwise)	-1.6590	-1.6610	-1.6590	-1.6613	-1.6571
D2019m03 (1 if March 2019; 0 otherwise)	1.6305	1.6413	1.5994	1.6417	1.6505
**COVID-19 (1 if April 2020 through September 2020; 0 otherwise)**	0.0210	0.0339	0.0399	0.0330	-0.0037
**Changes in Program Eligibility/ Administration**					
D2004M11	-0.0738	-0.0740	-0.0730	-0.0740	-0.0725
D2005M09	0.0811	0.0809	0.0802	0.0811	0.0802
D2005M11	0.0775	0.0767	0.0774	0.0764	0.0762
D2005M12	-0.0967	-0.0990	-0.0959	-0.0991	-0.1002
D2008M09	0.0748	0.0768	0.0735	0.0762	0.0731
D2017M11	-0.1134	-0.1136	-0.1133	-0.1135	-0.1135
**Lag of Dependent Variable (t-1)**	0.9821	0.9889	0.9633	0.9893	0.9923
**Lag Distribution of Macroeconomic Variable**					
Time Period (t)	-0.0302	0.00165	-0.0007	0.0016	0.0115
Time Period (t-1)	-0.0302	0.00165	-0.0013	0.0016	0.0115
Time Period (t-2)	NA	NA	-0.0018	NA	NA
Time Period (t-3)	NA	NA	-0.0021	NA	NA
Time Period (t-4)	NA	NA	-0.0024	NA	NA
Time Period (t-5)	NA	NA	-0.0026	NA	NA
Time Period (t-6)	NA	NA	-0.0026	NA	NA
Time Period (t-7)	NA	NA	-0.0026	NA	NA
Time Period (t-8)	NA	NA	-0.0024	NA	NA
Time Period (t-9)	NA	NA	-0.0021	NA	NA
Time Period (t-10)	NA	NA	-0.0018	NA	NA
Time Period (t-11)	NA	NA	-0.0013	NA	NA
Time Period (t-12)	NA	NA	-0.0007	NA	NA
**Sum of Lags**	-0.0605	0.0033	-0.0243	0.0031	0.0230
**Error Structure**					
AR(1)	0.5613	0.6204	0.6029	0.6161	0.5761
MA(1)	0.3053	0.3042	0.3296	0.3027	0.2369
**Constant**	1.0055	0.1931	0.8097	0.1867	-0.1574

Source: Econometric analysis done using EVIEWS 11.0.

The key metrics of the econometric distributed lag models concerning SNAP participation are exhibited in Table 2. The goodness-of-fit measures, R^2^ and adjusted R^2^, for the respective models range from 0.9989 to 0.9991, indicative of exceptional fit. In fact, we virtually explain all the variability in SNAP participation over the period October 1999 to September 2020. Holding all other factors invariant, on the basis of the respective models, SNAP participation rose 2.13 percent to 4.08 percent due to the presence of COVID-19. The impact of COVID-19 based on the initial claims of unemployment insurance was not statistically different from zero.

**Table 2:** Key metrics of the econometric distributed lag models concerning SNAP Participation by macroeconomic factor.

Macroeconomic Factor	Goodness-of-Fit R^2^	Goodness-of-Fit Adjusted R^2^	Durbin- Watson Statistic	Durbin h Statistic	Short-Run Elasticity	Long-Run Elasticity	Length of the Lag	Coefficient Associated with COVID-19
Initial Claims of	0.9991	0.9990	1.9409	0.4714	0.0115	0.0230	1	-0.00367
Unemployment Insurance					p-value = .0013	p-value = .0013		p-value = 0.7593
								-0.37
Manufactures’ New Orders of Durable Goods	0.9991	0.9990	1.9501	0.3982	-0.0302	-0.0605	1	0.0210
					p-value = .0122	p-value = .0122		p-value = .0001
								2.13
Housing Starts	0.9990	0.9989	1.9305	0.5454	-0.0007	-0.0243	12	0.0399
					p-value = .0336	p-value = .0336		p-value = .0000
								4.08
St Louis Financial Stress Index	0.9991	0.9990	1.9348	0.5203	0.0001	0.0002	1	0.0339
					p-value = .0010	p-value = .0010		p-value = .0000
								3.45
Kansas City Financial Stress Index	0.9989	0.9989	1.9457	0.4339	0.0003	0.0006	1	0.0330
					p-value = .0520	p-value = .0520		p-value = .0000
								3.36

Notes: (1) Elasticities for KC Financial Stress Index calculated at the sample means of the data. Elasticities for St. Louis Financial State Index calculated at the sample means of data. (2) The percentage change in the last column corresponds to (exp(coefficient associated with COVID-19)-1)*100.

Macroeconomic determinants of participation in the Supplemental Nutrition Assistance Program are: (1) the Kansas City and St. Louis Financial Stress Indices; (2) the number of initial claims for unemployment insurance; (3) manufacturers’ new orders of durable goods; and (4) housing starts. In particular, our findings confirm previous research done by the Economic Research Service (ERS) [41] which showed that the rise in SNAP participation was largely driven by economic conditions.

Importantly, we present the short-run and long-run elasticities associated with the macroeconomic factors on SNAP participation. These measures represent the sensitivity of SNAP participation due to a 1 percent change in any of the macroeconomic factors. To illustrate, the short-run and long-run elasticity associated with initial claims of unemployment insurance are 0.01148 and 0.02295. Hence a 10 percent change in initial claims of unemployment insurance leads to a 0.1148 percent change in SNAP participation in the short run and to a 0.2295 percent change in SNAP participation in the long run. The length of the lag in this dynamic process is 1 month. That is to say changes in SNAP participation due to changes in initial claims of unemployment insurance are not felt all at once but are distributed over time in the current month and over the past month.

The short-run elasticities of macroeconomic factors range from -0.03024 (manufacturers’ new orders of durable goods) to 0.01148 (initial claims of unemployment insurance). The long-run or cumulative elasticities of macroeconomic factors range from -0.06049 (manufacturers’ new orders of durable goods) to 0.02295 (inital claims of unemployment insurance). The length of the lag is 1 month for initial claims of unemployment insurance, manufacturers’ new orders of durable goods, and the respective financial stress indices, but 12 months for housing starts. Bottom line, changes in macroeconomic conditions concerning SNAP participation are not just contemporaneous but affect SNAP participation up to 12 months later.

#### Predictive analysis for SNAP participation

The actual and forecasted levels of SNAP participation over the period October 2020 to August 2021, along with the concomitant absolute percent errors are presented in Table 3. Most of the absolute percent errors are less than five percent across the respective models. The MAPE ranges from 3.5548 percent (initial claims of unemployment insurance) to 4.2617 percent (Kansas City Financial Stress Index). In terms of predictive ability of SNAP participation over the period Ocotober 2020 to August 2021, the initial claims for unemployment insurance is the best macroeconomic indicator based on the minimization of MAPE. That said, all of the five models perform well in mimicking the temporal dynamics of the level of SNAP participation.

**Table 3:** Out-of-Sample predictive ability of the distributed lag models concerning SNAP participation by macroeconomic factor.

			Manufacturers’ New Orders of Durable Goods	Housing Starts	Initial Claims of Unemployment Insurance	KC Stress Index	St Louis Stress Index
**Month**	**Year**	** Actual**			**Predicted SNAP Participation**		
10	2020	40,458,562	42,960,653	43,000,790	42,827,602	43,043,894	43,015,298
11	2020	40,788,366	42,387,265	42,514,786	42,134,263	42,563,872	42,492,507
12	2020	42,075,714	42,018,978	42,229,895	41,773,944	42,250,037	42,192,218
1	2021	43,805,208	41,944,928	42,224,654	41,789,973	42,224,382	42,204,186
2	2021	44,725,901	42,821,774	43,141,324	42,692,127	43,183,666	43,161,276
3	2021	44,515,528	42,946,484	43,278,361	42,748,201	43,345,604	43,335,663
4	2021	43,030,207	42,663,134	42,968,616	42,295,446	43,045,227	43,048,606
5	2021	41,102,832	43,120,664	43,408,755	42,513,534	43,506,076	43,524,061
6	2021	41,859,419	42,894,410	43,198,701	42,130,303	43,311,345	43,327,964
7	2021	40,725,960	44,301,426	44,577,322	43,478,887	44,759,323	44,765,878
8	2021	40,476,787	42,770,986	43,074,382	41,920,248	43,222,093	43,228,363
**Month**	**Year**		**Absolute Percent Error**				
10	2020		6.18	6.28	5.86	6.39	6.32
11	2020		3.92	4.23	3.30	4.35	4.18
12	2020		0.13	0.37	0.72	0.41	0.28
1	2021		4.25	3.61	4.60	3.61	3.65
2	2021		4.26	3.54	4.55	3.45	3.50
3	2021		3.52	2.78	3.97	2.63	2.65
4	2021		0.85	0.14	1.71	0.03	0.04
5	2021		4.91	5.61	3.43	5.85	5.89
6	2021		2.47	3.20	0.65	3.47	3.51
7	2021		8.78	9.46	6.76	9.90	9.92
8	2021		5.67	6.42	3.57	6.78	6.80
		MAPE	4.09	4.15	3.55	4.26	4.25

### Empirical results for WIC participation analysis

The estimated econometric models associated with WIC participation are exhibited in Table 4. The residuals move as an autoregressive process of order 1 and order 3 (AR(1)and AR(3)). To account for the AR(1) and the AR(3) processes associated with the residuals, we use generalized least squares (maximum likelihood) to obtain estimates of the coefficients in each of the respective models. Similar to the econometric analysis of SNAP participation, the signs of the coefficients associated with the macroeconomic factors are in agreement with prior expectations. Similar to the situation concerning SNAP participation, the significance of the one-month lag of the dependent variable indicates the presence of inertia or habitual behavior in the level of WIC participation.

**Table 4:** Estimated econometric models associated with WIC participation, October 1999 to September 2020.

	**Macroeconomic Variable**			
	**Real Disposable Personal Income**	**Ratio of Total Consumer Credit Owed to Disposable Personal Income**	**St. Louis Financial Stress Index**	**Real M2 Money Stock**
**Explanatory Variable**	**Estimated Coefficient**			
**Trend**				
Linear Trend (t = 0,1,2 …)	NA	NA	0.0002	0.0003
Quadratic Trend (Trend * Trend)	NA	-1.73E-07	-9.02E-07	-6.83E-07
**COVID-19 (1 if April 2020 through September 2020; 0 otherwise)**	0.0047	0.0063	0.0050	0.0060
**Seasonality**				
January (1 if month = January; 0 otherwise)	0.0228	0.0229	0.0228	0.0229
February (1 if month = February; 0 otherwise)	0.0009	0.0006	0.0014	0.0008
March (1 if month = March; 0 otherwise)	0.0242	0.0242	0.0241	0.0236
April (1 if month = April; 0 otherwise)	0.0122	0.0122	0.0124	0.0129
May (1 if month = May; 0 otherwise)	0.0170	0.0170	0.0174	0.0171
June (1 if month = June; 0 otherwise)	0.0164	0.0164	0.0169	0.0165
July (1 if month = July; 0 otherwise)	0.0132	0.0133	0.0138	0.0142
August (1 if month = August; 0 otherwise)	0.0221	0.0222	0.0230	0.0227
September (1 if month = September; 0 otherwise)	0.0093	0.0094	0.0102	0.0102
October (1 if month = October; 0 otherwise)	0.0148	0.0150	0.0154	0.0155
November (1 if month = November; 0 otherwise)	-0.0012	-0.0013	-0.0002	-0.0005
**Lag of Dependent Variable (t-1)**	1.0010	0.9981	0.9616	0.9652
**Lag Distribution of Macroeconomic Variable**				
Time Period (t)	-0.0025	0.0027	0.0005	-0.0015
Time Period (t-1)	-0.0040	0.0045	0.0005	-0.0027
Time Period (t-2)	-0.0045	0.0054	NA	-0.0037
Time Period (t-3)	-0.0040	0.0054	NA	-0.0045
Time Period (t-4)	-0.0025	0.0045	NA	-0.0050
Time Period (t-5)	NA	0.0027	NA	-0.0053
Time Period (t-6)	NA	NA	NA	-0.0055
Time Period (t-7)	NA	NA	NA	-0.0053
Time Period (t-8)	NA	NA	NA	-0.0050
Time Period (t-9)	NA	NA	NA	-0.0045
Time Period (t-10)	NA	NA	NA	-0.0037
Time Period (t-11)	NA	NA	NA	-0.0027
Time Period (t-12)	NA	NA	NA	-0.0015
Sum of Lags	-0.0173	0.0252	0.0011	-0.0506
**Error Structure**				
AR(1)	-0.2856	-0.3209	-0.3296	-0.3223
AR(3)	0.4350	0.4023	0.3791	0.3862
**Constant**	0.1330	-0.0594	0.5925	0.9371
	**Macroeconomic Variable**			
	**Housing Starts**	**Kansas City Financial Stress Index**	**Consumer Sentiment**	**Manufacturers’ New Orders of Durable Goods**
**Explanatory Variable**	**Estimated Coefficient**			
**Trend**				
Linear Trend (t = 0,1,2 …)	0.0003	0.0003	0.0002	0.0002
Quadratic Trend (Trend * Trend)	-1.58E-06	-1.46E-06	-1.12E-06	-8.79E-07
**COVID-19 (1 if April 2020 through September 2020; 0 otherwise)**	0.0062	0.0057	0.0038	0.0044
**Seasonality**				
January (1 if month = January; 0 otherwise)	0.0221	0.0225	0.0225	0.0226
February (1 if month = February; 0 otherwise)	0.0011	0.0005	0.0006	0.0010
March (1 if month = March; 0 otherwise)	0.0232	0.0232	0.0233	0.0239
April (1 if month = April; 0 otherwise)	0.0120	0.0125	0.0128	0.0122
May (1 if month = May; 0 otherwise)	0.0168	0.0167	0.0170	0.0170
June (1 if month = June; 0 otherwise)	0.0166	0.0166	0.0169	0.0166
July (1 if month = July; 0 otherwise)	0.0137	0.0137	0.0140	0.0135
August (1 if month = August; 0 otherwise)	0.0229	0.0227	0.0229	0.0227
September (1 if month = September; 0 otherwise)	0.0107	0.0104	0.0105	0.0100
October (1 if month = October; 0 otherwise)	0.0158	0.0159	0.0160	0.0154
November (1 if month = November; 0 otherwise)	0.0005	-0.0002	-0.0002	-0.0002
**Lag of Dependent Variable (t-1)**	0.9156	0.9385	0.9368	0.9624
**Lag Distribution of Macroeconomic Variable**				
Time Period (t)	-0.0035	7.4E-05	-0.0009	-0.0033
Time Period (t-1)	-0.0035	0.00013	-0.0016	-0.0033
Time Period (t-2)	NA	0.00017	-0.0020	NA
Time Period (t-3)	NA	0.00020	-0.0023	NA
Time Period (t-4)	NA	0.00021	-0.0024	NA
Time Period (t-5)	NA	0.00020	-0.0023	NA
Time Period (t-6)	NA	0.00017	-0.0020	NA
Time Period (t-7)	NA	0.00013	-0.0016	NA
Time Period (t-8)	NA	7.4E-05	-0.0009	NA
Time Period (t-9)	NA	NA	NA	NA
Time Period (t-10)	NA	NA	NA	NA
Time Period (t-11)	NA	NA	NA	NA
Time Period (t-12)	NA	NA	NA	NA
Sum of Lags	-0.0070	0.0014	-0.0160	-0.0066
**Error Structure**				
AR(1)	-0.2970	-0.3157	-0.3151	-0.2983
AR(3)	0.4008	0.3854	0.3894	-0.4156
**Constant**	1.3695	0.9526	1.0562	0.6564

The key metrics of the econometric distributed lag models concerning WIC participation are exhibited in Table 5. The goodness-of-fit measures, R^2^ and adjusted R^2^, for the respective models range from 0.9972 to 0.9976, indicative of exceptional fit. In fact, we virtually explain all the variability in WIC participation over the period October 1999 to September 2020. Holding all other factors invariant, on the basis of the respective models, WIC participation rose 0.38 percent to 0.63 percent due to the presence of COVID-19. WIC participation is higher in all months but for February and November relative to December. For February and November, no differences in WIC participation are evident relative to December.

**Table 5:** Key metrics of the econometric distributed lag models concerning WIC participation by macroeconomic factor.

Macroeconomic Factor	Goodness-of-Fit R^2^	Goodness-of-Fit Adjusted R^2^	Durbin-Watson Statistic	Durbin h Statistic	Short-Run Elasticity	Long-Run Elasticity	Length of the Lag	Coefficient Associated with COVID-19
Ratio of Consumer Credit to DPI	0.9975	0.9973	1.9851	0.1170	0.0027	0.0252	5	0.0063
					p-value = .0594	p-value = .0594		p-value = .0122
Real M2 Money Stock	0.9976	0.9974	1.9985	0.0119	-0.0015	-0.0506	12	0.0060
					p-value = .0049	p-value = .0049		p-value = .0116
								0.60
Consumer Sentiment	0.9976	0.9974	1.9847	0.1230	-0.0009	-0.0160	8	0.0038
					p-value = .0018	p-value = .0018		p-value = .1183
								0.38
Housing Starts	0.9976	0.9974	1.9625	0.3166	-0.0035	-0.0070	1	0.0062
					p-value = .0020	p-value = .0020		p-value = .0067
								0.62
St Louis Financial Stress Index	0.9976	0.9974	2.0015	-0.0123	3.43E-06	6.92E-05	1	0.0050
					p-value = .0002	p-value = .0002		p-value = .0480
								0.50
Kansas City Financial Stress Index	0.9976	0.9974	1.9803	0.1586	1.40.3E-005	0.0003	8	0.0057
					p-value = .0014	p-value = .0014		p-value = .0162
								0.57
Real Disposable Personal Income	0.9974	0.9972	1.9718	0.2228	-0.0025	-0.0173	4	0.0047
					p-value = .0000	p-value = .0000		p-value = .0701
								0.47
Manufacturers’ New Orders of Durable Goods	0.9975	0.9973	1.9793	0.1683	-0.0033	-0.0066	1	0.0044
					p-value = .0839	p-value = .0839		p-value = .0783
								0.45

Notes: (1) Elasticities for KC Financial Stress Index calculated at the sample means of the data. Elasticities for St. Louis Financiial Stress Index calculated at the sample means of the data. (2) The percentage change in the last column corresponds to (exp(coefficient associated with COVID-19)-1)*100.

Macroeconomic drivers of the Special Supplemental Nutrition Program for Women, Infants, and Children are: (1) the Kansas City and St. Louis Financial Stress Indices; (2) real disposable personal income; (3) real M2 money stock; (4) housing starts; (5) consumer sentiment; (6) the ratio of total consumer credit owed to disposable personal income; and (7) manufacturers’ new orders of durable goods.

Importantly, we present the short-run and long-run elasticities associated with the macroeconomic factors on WIC participation. These measures represent the sensitivity of WIC participation due to a 1 percent change in any of the macroeconomic factors. To illustrate, the short-run and long-run elasticity associated with the ratio of total consumer credit to disposable personal income are 0.00270 and 0.02517. Hence a 10 percent change in this ratio results in a 0.0027 percent change in WIC participation in the short run and to a 0.2517 percent change in WIC participation in the long run. The length of the lag in this dynamic process is 5 months. That is to say changes in WIC participation due to changes in the ratio of total consumer credit to disposable personal income are not felt all at once but are distributed over time in the current month and five months later.

The short-run elasticities of macroeconomic factors range from -0.00332 to 0.00270 (ratio of total consumer credit to disposable personal income). The long-run or cumulative elasticities of macroeconomic factors range from -0.05059 (real M2 money stock) to 0.02517 (ratio of total consumer credit to disposable personal income). The length of the lag is one month for housing starts, the St. Louis Financial Stress Index, and manufacturers’ new orders of durable goods. Further, length of the lag is 4 months for real disposable personal income, 5 months for the ratio of total consumer credit to disposable personal income. 8 months for consumer sentiment and the Kansas City Financial Stress Index respectively, and 12 months for real M2 money stock. Bottom line, changes in macroeconomic conditions affect WIC participation not just contemporaneously but also influence WIC participation up to 12 months later.

#### Predictive analysis for WIC participation

The actual and forecasted levels of WIC participation over the period October 2020 to August 2021, along with the concomitant absolute percent errors, are presented in Table 6. Across the respective models, all absolute percent errors over the period are less than five percent. The MAPE ranges from 1.2016 percent (ratio of total consumer credit to disposable personal income) to 3.6138 percent (real M2). In terms of predictive ability of WIC participation over the period October 2020 to August 2021, the ratio of total consumer debt to disposable personal income, consumer sentiment, and real disposable personal income are the best macroeconomic indicators based on the minimization of MAPE. That said, all of the eight previously mentioned models perform well in imitating the temporal dynamics of the level of WIC participation.

**Table 6:** Out-of-Sample predictive ability of WIC participation distributed lag models by macroeconomic factor.

			Consumer Sentiment	Ratio of Total Consumer Debt To DPI	Real DPI	Durable Goods	Housing Starts	KC Stress Index	Real M2	St Louis Stress Index
**Month**	**Year**	**Actual**	**Predicted**							
10	2020	6,381,064	6,357,027	6,344,041	6,351,967	6,336,855	6,330,036	6,343,661	6,323,206	6,334,448
11	2020	6,331,773	6,245,624	6,227,342	6,235,952	6,212,945	6,201,576	6,222,335	6,188,332	6,212,870
12	2020	6,330,413	6,185,509	6,166,524	6,179,709	6,141,469	6,121,766	6,150,029	6,099,690	6,138,282
1	2021	6,315,085	6,236,066	6,209,792	6,229,357	6,172,505	6,144,486	6,184,054	6,109,743	6,169,103
2	2021	6,261,639	6,146,572	6,119,683	6,142,512	6,072,205	6,042,135	6,082,378	5,990,120	6,073,094
3	2021	6,299,315	6,216,739	6,187,267	6,217,941	6,131,229	6,094,844	6,137,295	6,020,713	6,131,094
4	2021	6,239,999	6,196,829	6,156,998	6,195,308	6,094,307	6,051,698	6,102,414	5,963,219	6,095,720
5	2021	6,170,818	6,207,188	6,163,017	6,208,070	6,094,678	6,048,103	6,099,070	5,939,951	6,098,561
6	2021	6,151,239	6,217,718	6,168,971	6,226,893	6,095,281	6,042,423	6,094,699	5,913,554	6,098,264
7	2021	6,134,929	6,198,878	6,148,484	6,215,888	6,065,233	6,008,538	6,061,864	5,864,676	6,069,993
8	2021	6,146,528	6,238,764	6,190,776	6,269,796	6,096,166	6,036,456	6,086,813	5,871,279	6,102,372
**Month**	**Year**		**Absolute Percent Error**							
10	2020		0.38	0.58	0.46	0.69	0.80	0.59	0.91	0.73
11	2020		1.36	1.65	1.51	1.88	2.06	1.73	2.27	1.88
12	2020		2.29	2.59	2.38	2.98	3.30	2.85	3.64	3.04
1	2021		1.25	1.67	1.36	2.26	2.70	2.07	3.25	2.31
2	2021		1.84	2.27	1.90	3.03	3.51	2.86	4.34	3.01
3	2021		1.31	1.78	1.29	2.67	3.25	2.57	4.42	2.67
4	2021		0.69	1.33	0.72	2.33	3.02	2.20	4.44	2.31
5	2021		0.59	0.13	0.60	1.23	1.99	1.16	3.74	1.17
6	2021		1.08	0.29	1.23	0.91	1.77	0.92	3.86	0.86
7	2021		1.04	0.22	1.32	1.14	2.06	1.19	4.41	1.06
8	2021		1.50	0.72	2.01	0.82	1.79	0.97	4.48	0.72
		MAPE	**1.21**	**1.20**	**134**	**1.81**	**2.38**	**1.74**	**3.61**	**1.80**

### Empirical results for NSLP participation analysis

The estimated econometric models associated with NSLP participation are exhibited in Table 7. We capture seasonality through the use of three dummy variables corresponding to the month of June, July, and August. Unlike the models for SNAP participation and WIC participation, in the NSLP models, no lag of the dependent variable is present. Additionally, the residuals follow a moving average process of order 12. To account for the error structure associated with the residuals, we use generalized least squares (maximum likelihood) to obtain estimates of the coefficients in each of the respective models. Similar to the econometric analysis of SNAP participation and WIC participation, the signs of the coefficients associated with the macroeconomic factors are in agreement with prior expectations.

**Table 7:** Estimated econometric models associated with NSLP participation, October 1999 to September 2020.

Explanatory Variable	Unemploymnet Rate	Initial Claims of Unemployment Insurance
	*Estimated Coefficient*	
**COVID-19 (1 if April 2020 through September 2020; 0 otherwise)**	-1.1683	-1.3246
**Seasonality**		
June (1 if month = June; 0 otherwise)	-0.8877	-0.8885
July (1 if month = July; 0 otherwise)	-3.0010	-3.0041
August (1 if month = August; 0 otherwise)	-0.4883	-0.4881
**Lag Distribution of Macroeconomic Variable**		
Time Period (t)	0.0337	0.0246
Time Period (t-1)	0.0506	0.0393
Time Period (t-2)	0.0506	0.0442
Time Period (t-3)	0.0337	0.0393
Time Period (t-4)	NA	0.0246
Sum of Lags	0.1687	0.1718
**Error Structure**		
MA(12)	0.8213	0.8003
**Constant**	16.9160	15.0213

The key metrics of the econometric distributed lag models concerning NSLP participation are exhibited in Table 8. The goodness-of-fit measures, R^2^ and adjusted R^2^, for the respective models range from 0.9832 to 0.9837, once again indicative of exceptional fit. In fact, we virtually explain all the variability in NSLP participation over the period October 1999 to September 2020.

**Table 8:** Key metrics of the econometric distributed lag models concerning NSLP participation by macroeconomic factor.

Macroeconomic Factor	Goodness-of-Fit R^2^	Goodness-of-Fit Adjusted R^2^	Durbin-Watson Statistic	Short-Run Elasticity	Long-Run Elasticity	Length of the Lag	Coefficient Associated with COVID-19
Initial Claims of Unemployment Insurance	0.9837	0.9832	1.99	0.0246	0.17183	4	-1.3246
				p-value = .0001	p-value = .0001		p-value = .0000
							-73.41%
Unemployment Rate	0.9837	0.9832	2.04	0.0337	0.1687	1	-1.1683
				p-value = .0014	p-value = .0014		p-value = .0000
							-68.91%

Note: The percentage change in the last column corresponds to (exp(coefficient associated with COVID-19)-1)*100.

Holding all other factors invariant, based on the respective models, NSLP participation fell 68.91 percent and 73.41 percent due to the presence of COVID-19. This result likely is primarily due to restrictions on attending school during the COVID-19 pandemic. Macroeconomic factors affecting the number of participants in the National School Lunch Program are: (1) the number of initial claims for unemployment insurance; and (2) the unemployment rate. The number of macroeconomic determinants associated with NSLP participation is far fewer than the number of macroeconomic determinants associated with the SNAP and the WIC program.

We present the short-run and long-run elasticities associated with the macroeconomic factors on NSLP participation. These measures represent the sensitivity of NSLP participation due to a 1 percent change in any of the macroeconomic factors. To illustrate, the short-run and long-run elasticity associated with the unemployment rate are 0.03374 and 0.1687 repectively. Hence a 10 percent change in the unemployment rate results in a 0.3374 percent change in NSLP participation in the short run and to a 1.687 percent change in NSLP participation in the long run. The length of the lag in this dynamic process is 3 months. That is to say changes in NSLP participation due to changes in consumer sentiment are not felt all at once but are distributed over time not only in the current month but also the previous three months.

The short-run elasticities of macroeconomic factors range from -0.2455 (initial claims of unemployment insurance) to 0.03374 (unemployment rate). The long-run or cumulative elasticities of macroeconomic factors range from 0.1687 (unemployment rate) to 0.17183 (iniital claims of unemployment insurance). The length of the lag is three months for the unemployment rate, and the lag length is four months for initial claims of unemployment insurance. Bottom line, changes in macroeconomic conditions affect NSLP participation not just contemporaneously but also influence NSLP participation up to four months later.

#### Predictive analysis for NSLP participation

The actual and forecasted levels of NSLP participation over the period October 2020 to August 2021, along with the concomitant absolute percent errors, are presented in Table 9. For the NSLP models, the MAPE ranges from 21.19 percent (initial claims of unemployment insurance) to 21.48 percent (the unemployment rate). In terms of predictive ability of NSLP participation over the period October 2020 to August 2021, the respective models perform well in mimicking the temporal dynamics of the level of NSLP participation, especially given the upheaval associated with the pandemic and school attendance.

**Table 9:** Out-of-Sample predictive ability of NSLP distributed lag models by macroeconomic factor.

			Initial Claims of Unemployment Insurance	Umemployment Rate
**Month**	**Year**	**Actual**	**Predicted**	**Predicted**
10	2020	8,397,041	9,901,144	10,269,188
11	2020	8,365,658	9,646,526	10,083,570
12	2020	7,870,890	9,474,698	9,967,342
1	2021	7,963,962	9,468,493	9,924,922
2	2021	8,592,083	9,555,577	9,969,844
3	2021	9,386,129	9,765,887	10,594,596
4	2021	9,945,311	6,655,152	6,595,380
5	2021	9,879,058	5,747,990	5,962,866
6	2021	4,865,792	3,308,260	3,625,181
7	2021	1,564,353	1,251,668	1,460,264
8	2021	5,337,821	4,351,093	4,922,452
**Month**	**Year**	**Absolute Percent Error**		
10	2020		17.91	22.30
11	2020		15.31	20.54
12	2020		20.38	26.64
1	2021		18.89	24.62
2	2021		11.21	16.04
3	2021		4.05	12.88
4	2021		33.08	33.68
5	2021		41.82	39.64
6	2021		32.01	25.50
7	2021		19.99	6.65
8	2021		18.49	7.78
		MAPE	21.19	21.48

## Conclusions

We identify and assess the importance of macroeconomic factors on participation in key food assistance programs administered by the FNS over the period October 1999 to September 2020. Macroeconomic determinants of participation in SNAP are: (1) the Kansas City and St. Louis Financial Stress Indices; (2) the number of initial claims for unemployment insurance; (3) manufacturers’ new orders of durable goods; (4) housing starts; and (5) consumer sentiment. Macroeconomic drivers of WIC are: (1) the Kansas City and St. Louis Financial Stress Indices; (2) real disposable personal income; (3) real M2 money stock; (4) housing starts; (5) consumer sentiment; (6) the ratio of total consumer credit owed to disposable personal income; and (7) manufacturers’ new orders of durable goods. Macroeconomic factors affecting the number of participants in the NSLP are: (1) the number of initial claims for unemployment insurance; and (2) the unemployment rate.

Different sets of macroeconomic drivers which affect the respective food assistance programs are evident. No macroeconomic factor is common across SNAP, WIC, and NSLP participation. In addition, manufacturers’ orders of non-defense capital goods and the Case-Shiller Home Price Index play no statistically significant role in affecting participation levels of the SNAP, WIC, and NSLP. As well, changes in macroeconomic conditions which influence SNAP, WIC and NSLP participation are not only contemporaneous but also affect participation levels anywhere from 1 month to 12 months later.

This work confirms that the impacts associated with the Kansas City Financial Stress Index; the St. Louis Financial Stress Index; the number of initial claims for unemployment insurance; the unemployment rate; and the ratio of total consumer credit outstanding to disposable personal income are positively related to the level of participation in the SNAP, WIC, and NSLP. Further, this research substantiates that the impacts associated with manufacturers’ new orders of durable goods; real disposable personal income; real M2 money stock; housing starts; and the University of Michigan Consumer Sentiment Index are negatively related to the level of participation in the SNAP, WIC, and NSLP. Importantly, we quantify the impact of COVID-19 on participation in the SNAP, WIC, and NSLP. The structural/econometric models mimic well the level of participation in SNAP, WIC, and the NSLP over the period October 2020 to August 2021 on the basis of mean absolute percent error.

This study is useful for government policy makers in considering participation in key nutrition and food assistance programs adjusting for macroeconomic shocks. With knowledge of the marginal effects (and elasticities) as well as predictions associated with participation rates in SNAP, WIC, and the NSLP based on the aforementioned macroeconomic drivers, policy makers will be in better position to assess program costs and to minimize errors in the budgetary process. The extant literature places emphasis primarily on the level of real income, the volatility of income, and the unemployment rate as the key macroeconomic variables in affecting participation in domestic nutrition assistance programs. Unlike previous studies, we find that a granular array of other macroeconomic variables also affects participation levels in these programs. Importantly, we expand coverage to WIC and NSLP participation and not just center attention on SNAP participation.

Because we quantify the impact of COVID-19 on level of participation in SNAP, WIC, and the NSLP, we provide knowledge to analysts that was heretofore lacking to help them better prepare for future pandemics or other major shocks to the economy. As well, for the first time in the extant literature, this analysis deals with the use of polynomial distributed lags in econometric models to allow for dynamics of macroeconomic shocks in affecting levels of participation in SNAP, WIC, and the NSLP. We also capture inertia with the use of lags of participation levels. Unequivocally, this research adds measurably to the extant economic literature on this topic.

In the ex-ante period from October 2020 to August 2021, the MAPE was lowest for the WIC analysis, ranging from 1.20 percent to 1.83 percent compared to the SNAP analysis, varying from 3.55 percent to 4.26 percent. However, the actual number of SNAP participants were roughly seven times larger than the number of WIC participants. On the other hand, the MAPE was highest for the NSLP analysis, ranging from 21.2 percent to 21.5 percent. The actual number of NSLP participants varied from 1.5 million to nearly 10.0 million during the ex-ante period. But the variability associated with the number of participants in the NSLP program exceeded by far the variability in the number of participants in the SNAP and WIC programs. FNS staff and analysts should consider the econometric modeling efforts to reduce forecasting errors, especially regarding the level of SNAP participation and the level of WIC participation. In this way, the likelihood of budgeting misallocations is diminished. This econometric analysis then provides a baseline for forecast accuracy. The added value associated with our forecasts rests on comparing the MAPE from these models to the MAPE of FNS analysts.

In updates to this research, we may examine empirically the effects of macroeconomic factors during the COVID-19 lockdown, thereby including interaction terms in the models. For future endeavors, we recommend replicating this analysis for various states and/or regions. Further, we recommend conducting this analysis for other major nutrition assistance programs such as the School Breakfast Program (SBP) and the Child and Adult Care Food Program (CACFP). The chief recommendation to the FNS is to consider the impacts of macroeconomic factors on participation in the various food assistance programs and to employ our polynomial distributed lag models for forecasting purposes.
